# Hyperinsulinemia-induced upregulation of adipocyte TPH2 contributes to peripheral serotonin production, metabolic dysfunction, and obesity

**DOI:** 10.1172/JCI190765

**Published:** 2025-05-30

**Authors:** Brian I. Park, Andrew R. Reeves, Ying Zhu, Robin A. Wilson, Sophia C. Fernandes, Kimberly K. Buhman, Kelli A. Lytle, Michael D. Jensen, Andrew S. Greenberg

**Affiliations:** 1 Jean Mayer USDA Human Nutrition Research Center on Aging and; 2 Gerald J. and Dorothy R. Friedman School of Nutrition Science and Policy, Tufts University, Boston, Massachusetts, USA.; 3 Department of Nutrition Science at Purdue University, West Lafayette, Indiana, USA.; 4 Endocrine Research Unit, Mayo Clinic, Rochester, Minnesota, USA.; 5 School of Medicine, Tufts University, Boston, Massachusetts, USA.

**Keywords:** Endocrinology, Metabolism, Adipose tissue, Insulin, Obesity

## Abstract

Tryptophan hydroxylase (TPH) is a rate-limiting enzyme for serotonin or 5-hydroxytryptamine (5-HT) synthesis. Previously, adipocyte TPH1 has been linked to increased adipose 5-HT, reduced brown adipose tissue (BAT) thermogenesis, and obesity. However, the role of TPH2, a neural isoform highly expressed in obese adipose tissue, is unknown. Here, we report that adipose tissue expression of TPH2 is dramatically elevated in mice with diet-induced obesity (DIO) and *ob/ob* mice, as well as in obese humans. In mice fed a high-fat diet, adipocyte TPH2 deficiency improved DIO-induced metabolic complications, enhanced BAT thermogenesis, and increased intestinal energy-harvesting efficiency without affecting adiposity. Conversely, TPH2 overexpression in epididymal adipocytes of chow-fed mice raised adipose and plasma 5-HT levels, suppressed BAT thermogenesis, and exacerbated obesity and metabolic dysfunction. We found that obesity-induced hyperinsulinemia upregulated adipocyte TPH2 expression via activation of mechanistic target of rapamycin complex 1 and SREBP1. In humans, *TPH2* mRNA levels in subcutaneous adipose tissue, but not those of *TPH1*, are positively correlated with fasting plasma insulin concentrations. In summary, our study demonstrates that obesity-associated increases in adipocyte TPH2 can regulate distal tissue physiology and energy metabolism, suggesting that TPH2 could be a potential therapeutic target for obesity and its associated complications.

## Abstract 2

Obese mice and humans exhibit increased expression of adipocyte tryptophan hydroxylase 2, elevating local and circulating serotonin levels, which promote obesity and metabolic complications.

## Introduction

Obesity and its associated medical complications are a major global health challenge, with their prevalence reaching epidemic proportions in many countries (1, 2). While various factors, such as a sedentary lifestyle and genetic differences, promote the development of obesity, chronic energy imbalance between calorie intake and energy expenditure (EE) is undoubtedly a major contributor (3). Diet-induced obesity (DIO) causes adipocyte dysfunction, which is strongly associated with the progression of obesity and the metabolic complications of cardiovascular disorders, liver disease, and type 2 diabetes mellitus (T2DM) (4, 5). Beyond being an essential reservoir of energy, adipocytes have an integral role in regulating systemic metabolism through a complex orchestration of adipokines and metabolites (6). Healthy adipocytes secrete beneficial adipokines (adiponectin) and lipid metabolites (palmitic acid hydroxystearic acids) that improve energy metabolism and obesity-associated metabolic complications (7–9). In contrast, adipocytes in the context of obesity become dysfunctional and produce molecules such as ceramides that act locally to dysregulate white and brown adipocyte metabolism and are secreted into the bloodstream, where they can promote hepatic steatosis (10, 11). Despite the pivotal role of adipocytes in the progression of obesity and its associated metabolic alterations, the underlying molecular mechanisms by which DIO causes adipocyte dysfunction and its role in metabolic health have not been completely elucidated.

While multiple factors contribute to the progression of DIO and its metabolic complications, a recent line of evidence highlighted the possible role of adipocyte-derived 5-hydroxytryptamine (5-HT) in regulating both adipocyte and systemic metabolisms (12). 5-HT is a biogenic monoamine that is synthesized from the amino acid tryptophan (13). The synthesis of 5-HT is tightly regulated by the availability of tryptophan, with its hydroxylation being the rate-limiting step catalyzed by tryptophan hydroxylase 1 (TPH1) and TPH2 (14). TPH1 is mainly expressed outside of the CNS, whereas TPH2 has been thought to be exclusively located in the CNS and enteric nervous system (15, 16). A major source for peripheral, circulating 5-HT is intestinal TPH1, which can modulate hepatic glucose and lipid metabolism (17). However, in mice fed a high-fat diet (HFD), gut-specific deletion of TPH1 did not alter adipose tissue weight, brown adipose tissue (BAT) thermogenesis, or systemic EE (18). Previous studies have found that in DIO mice, adipocyte expression of TPH1 is increased in white adipose tissue (WAT) and BAT, which led to increased local adipose tissue levels of 5-HT, but not in plasma (19, 20). Interestingly, genetic deletion of adipocyte TPH1 protected HFD-fed mice from DIO and its metabolic complications, most likely due to increased BAT thermogenesis and systemic EE (20). In addition to studies with TPH1-knockout mouse models, chemical inhibition of membrane-bound 5-HT receptor (5-HTR) signaling in the periphery also prevented mice from developing DIO, indicating that 5-HT promotes obesity via receptor-mediated signaling (17). Furthermore, a recent study reported that in the visceral adipose tissue of obese humans, the expression level of both serotonin receptors, 5-HTR2a and 5-HTR2b, was increased and positively correlated with BMI, alanine transaminase (ALT), and aspartate transaminase (AST) levels, suggesting a potential role for 5-HT signaling in human obesity (21). However, our understanding of adipocyte 5-HT production and its link to obesity in humans remains unclear.

While the metabolic role of adipocyte TPH1 has been investigated, the expression of TPH2, an isoform primarily expressed in neural cells, and its contribution to the development of DIO and associated metabolic complications remain unknown. Interestingly, previous studies have reported increased *Tph2* mRNA levels in the adipose tissues of obese mice; however, the metabolic role of adipocyte TPH2 has never been investigated (22, 23). Here, we demonstrate a role for adipocyte TPH2 in contributing to the development of DIO and metabolic complications. Notably, obese humans exhibit increased *TPH2* mRNA levels, but not *TPH1* mRNA levels, in subcutaneous adipose tissue, and these levels are positively correlated with plasma ALT and triglyceride (TG) levels. In HFD-fed mice, genetic deletion of adipocyte-specific TPH2 reduced both adipose and circulating levels of 5-HT, adipose depot weights, and improved glucose homeostasis and hepatic steatosis, while increasing systemic EE and intestinal energy-harvesting efficiency. Overexpression of TPH2 specifically in epididymal WAT (eWAT) adipocytes of chow-fed mice increased local adipose tissue and systemic levels of 5-HT, resulting in increased adiposity and the development of metabolic complications. We also found that incubating primary adipocytes with insulin robustly increased TPH2 expression, demonstrating that the dramatic upregulation of adipocyte TPH2 in obese mice is driven by obesity-induced hyperinsulinemia. Consistent with this observation, in human subcutaneous adipose tissue, *TPH2* mRNA levels were positively correlated with fasting insulin levels. These findings highlight the role of hyperinsulinemia-induced adipocyte TPH2 expression in regulating both local and distal organ physiology that can exacerbate obesity and its metabolic complications.

## Results

### DIO increases adipocyte 5-HT synthesis, and genetic ablation of adipocyte TPH2 improves glucose homeostasis.

**Figure 1:**
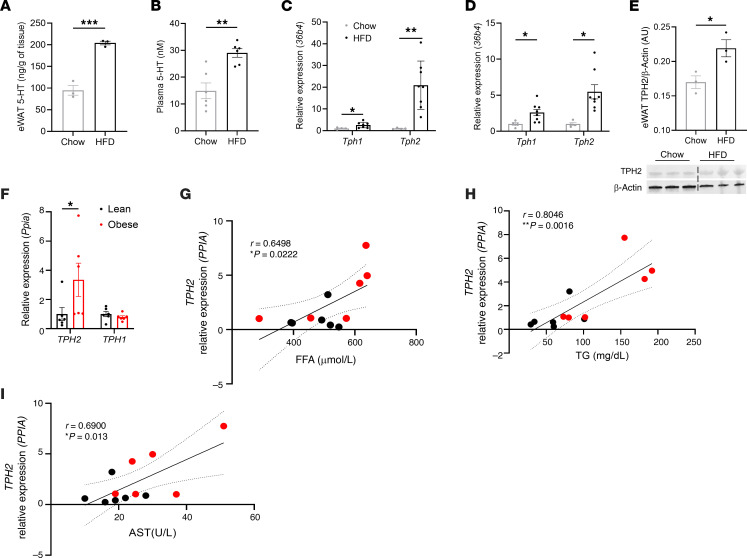
Obesity dramatically upregulates adipocyte TPH2 expression in both mice and humans. (**A**) eWAT 5-HT levels in chow- or HFD-fed mice for 6 weeks (*n* = 3 per group). (**B**) Plasma 5-HT levels in chow- or HFD-fed mice for 6 weeks (*n* = 6 per group). (**C**) mRNA levels of *Tph1* and *Tph2* of isolated epididymal white adipocytes in chow- or HFD-fed mice for 6 weeks (*n* = 4 for chow; *n* = 8 for HFD). (**D**) mRNA levels of *Tph1* and *Tph2* of isolated brown adipocytes in chow- or HFD-fed mice for 6 weeks (*n* = 4 for chow; *n* = 8 for HFD). (**E**) eWAT protein levels of TPH2 in chow- or HFD-fed mice for 6 weeks (*n* = 3 per group). (**F**) mRNA levels of *TPH1* and *TPH2* in human subcutaneous fat from lean and obese subjects (*n* = 6 per group). (**G**–**I**) Correlation between TPH2 expression in human subcutaneous fat and fasting plasma FFA (**G**),TG (**H**), and AST (**I**) levels of lean and obese individuals (*n* = 6 per group). Pearson’s *r* correlation coefficient with corresponding *P* values. Data are presented as mean ± SEM. For statistical analysis, 2-tailed Student’s *t* test (**A**–**F**) was used. *P < 0.05, **P < 0.01, ***P < 0.001.

We first investigated the levels of 5-HT in eWAT and plasma of chow- and HFD-fed mice and found that DIO increased both eWAT and circulating levels of 5-HT (Figure 1, A and B and Supplemental Figure 1A; supplemental material available online with this article; https://doi.org/10.1172/JCI190765DS1). To determine whether TPH2 contributed to the increased 5-HT levels in HFD-fed mice, we analyzed the mRNA levels of adipocyte *Tph1* and *Tph2* in chow- and HFD-fed mice and observed that HFD feeding increased expression of both genes in mature adipocytes from eWAT and BAT (Figure 1, C and D). DIO-induced upregulation of *Tph2* gene expression also resulted in a significant increase in eWAT TPH2 protein level (Figure 1E). To assess the potential translational significance of obesity-induced adipose TPH2 expression, we examined the expression of *TPH1* and *TPH2* in lean and obese humans. We observed that *TPH2* mRNA levels were higher in the subcutaneous fat of obese individuals, while *TPH1* mRNA levels were not different compared with those of lean subjects (Figure 1F). Since a recent study reported positive correlations between *5-Htr2* gene expression and liver function markers (21), we also assessed the relationship between *TPH2* mRNA levels in subcutaneous WAT and various blood parameters of both lean and obese individuals. Of note, plasma levels of free fatty acid (FFA), TG, and AST showed a strong positive correlation with *TPH2* gene expression levels (Figure 1, G–I), while other parameters, such as total cholesterol, LDL cholesterol, HDL cholesterol, and non-HDL were not correlated with subcutaneous WAT *TPH2* mRNA levels (Supplemental Figure 1, B–E). Taken together, these data suggest that adipocyte TPH2 in obese mice and humans may contribute to the peripheral role of 5-HT and obesity-associated metabolic complications.

**Figure 2:**
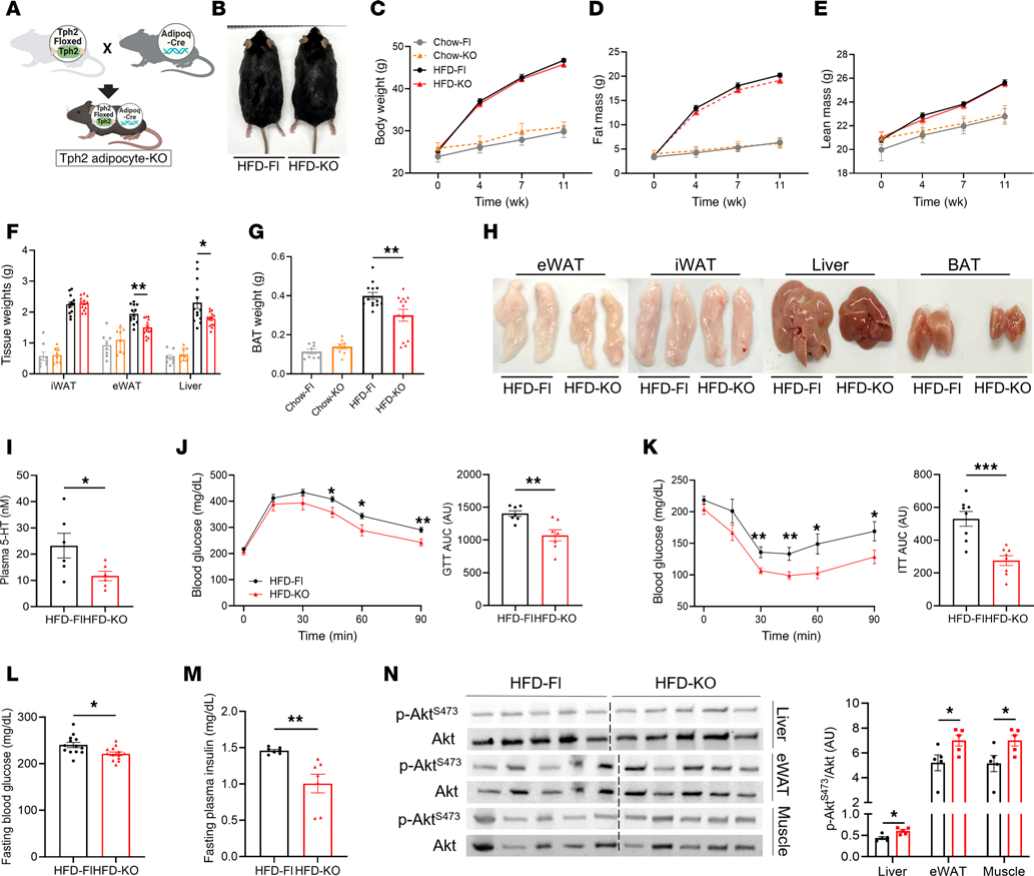
Mice with adipocyte-specific deficiency of TPH2 are not resistant to HFD-induced weight gain but have improved glucose tolerance and insulin sensitivity, and reduced circulating 5-HT levels. (**A**) Overview of generating adipocyte-specific TPH2-deficient mice (**B**) Representative photograph of HFD-Fl and HFD-KO mice after 12 weeks of HFD feeding. (**C**–**E**) Time course of body weight (**C**), fat (**D**), and lean mass (**E**) following HFD feeding (*n* = 8 for Chow-Fl and Chow-KO; *n* = 14 for HFD-Fl and HFD-KO). (**F**–**H**) Tissue weights of inguinal white adipose tissue (iWAT), eWAT, liver (**F**), and BAT (**G**), and representative photographs of collected tissues (**H**) (*n* = 8 for Chow-Fl and Chow-KO; *n* = 13 for HFD-Fl and HFD-KO). (**I**) Plasma levels of 5-HT from HFD-Fl and HFD-KO mice after 12 weeks of HFD feeding (*n* = 6 per group). (**J**) Glucose tolerance test (GTT) and AUC performed after 7 weeks of HFD feeding (*n* = 7 per group). (**K**) Insulin tolerance test (ITT) and AUC performed after 9 weeks of HFD feeding (*n* = 8 per group). (**L** and **M**) Fasting blood glucose (*n* = 13 per group) and plasma insulin levels (*n* = 7 per group) after 12 weeks of HFD feeding. (**N**) Relative levels of phosphorylated Akt (Ser473) to total Akt in liver, eWAT, and gastrocnemius muscle of HFD-Fl and HFD-KO mice, 15 minutes after an insulin injection following 8 weeks of HFD feeding (*n* = 5 per group). Data are presented as mean ± SEM. For statistical analysis, 2-way ANOVA with Dunnett’s test (**C**–**G**) or 2-tailed Student’s *t* test (**I**–**N**) was used. *P < 0.05, **P < 0.01, ***P < 0.001.

To comprehensively investigate the physiologic role of adipocyte TPH2 in vivo, we generated mice deficient in adipocyte-specific TPH2 expression by crossing TPH2^loxP/loxP^ with adiponectin (Adipoq)-Cre mice. TPH2^loxP/loxP^ mice with or without Adipoq-Cre were fed either chow or a HFD for 12 weeks (hereafter referred to as Chow-Fl, Chow-KO, HFD-Fl, and HFD-KO mice, respectively; Figure 2A). In all groups of mice, adipocyte *Tph2* expression was decreased (Supplemental Figure 1, F and G). While we did not observe statistically significant differences in total body weight and fat between different genotype groups fed chow or a HFD, the weights of eWAT, liver, and BAT were lower in HFD-KO mice compared with that of HFD-Fl mice (Figure 2, B–H). Unlike mice with TPH1 adipocyte deficiency (20), we found that genetic deletion of adipocyte TPH2 decreased the circulating 5-HT concentrations in HFD-fed mice (Figure 2I). Adipocyte ablation of TPH2 did not protect mice from HFD-induced weight gain; still, HFD-KO mice exhibited improved glucose homeostasis, as evidenced by enhanced glucose tolerance and insulin sensitivity compared with HFD-Fl mice (Figure 2, J and K). HFD-KO mice also had lower levels of fasting blood glucose and plasma insulin concentrations compared with HFD-Fl mice (Figure 2, L and M). Consistent with these observations, insulin-stimulated phosphorylation of Akt was increased in liver, eWAT, and muscle from HFD-KO mice compared with HFD-Fl mice (Figure 2N). Collectively, in DIO mice, inhibiting adipocyte expression of TPH2 improves obesity-induced glucose intolerance and insulin resistance.

### Loss of adipocyte TPH2 protected mice from DIO-induced hepatic steatosis and adipocyte dysfunction.

**Figure 3:**
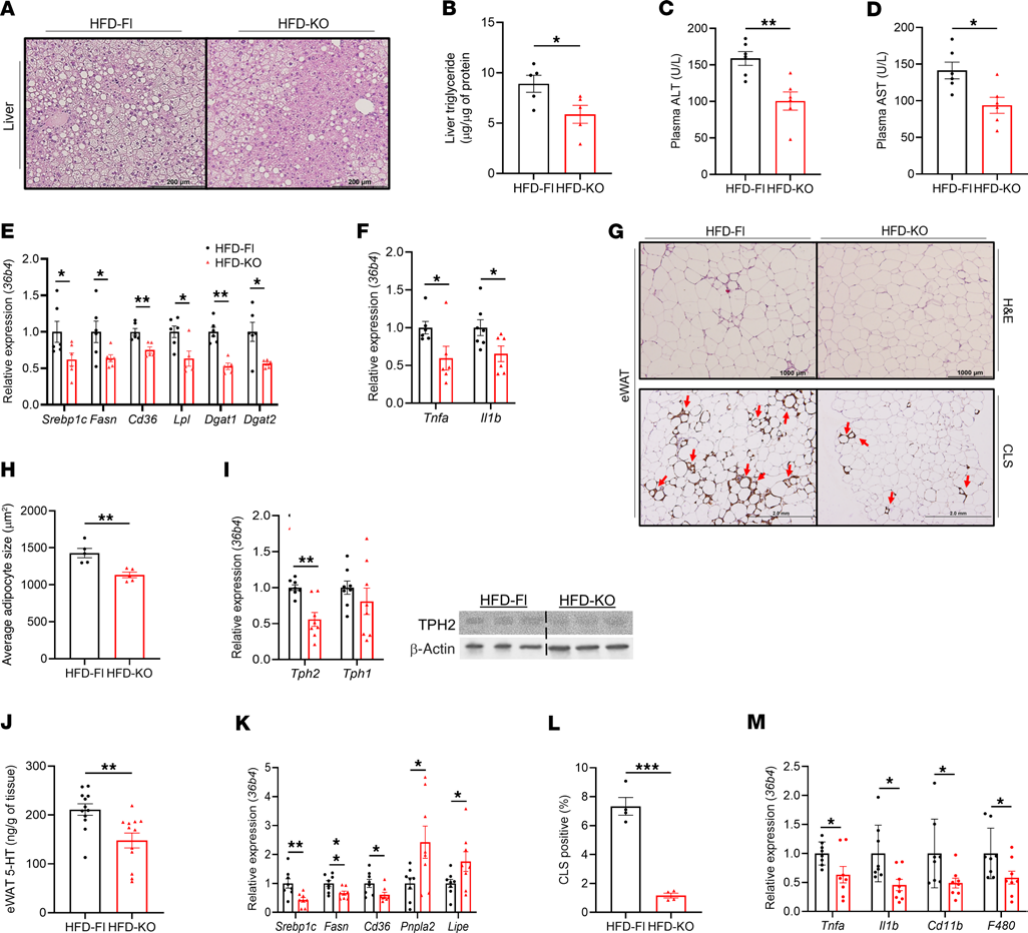
Genetic ablation of adipocyte TPH2 expression protects mice from HFD-induced hepatic steatosis and adipocyte dysfunction. (**A**) Representative images of H&E-stained liver after 12 weeks of HFD feeding (*n* = 5 per group). Scale bars: 200 μm. (**B**) TG levels in liver of mice after 12 weeks of HFD feeding (*n* = 5 per group). (**C** and **D**) Serum levels of ALT (**C**) and AST (**D**) (*n* = 5 per group). (**E** and **F**) mRNA levels of lipid metabolism–related genes (**E**) and proinflammatory genes (**F**) of liver after 12 weeks of HFD feeding (*n* = 6 per group). (**G**) Representative images of H&E staining and Galectin-3 (GAL-3 or MAC-2) immunohistochemical staining of eWAT showing CLS formation (indicated by red arrows) after 12 weeks of HFD feeding (*n* = 5 per group). Scale bars: 1,000 μm (top), 2 mm (bottom). (**H**) Average adipocyte size of mice after 12 weeks of HFD feeding (*n* = 5). (**I**) mRNA levels of *Tph2* and *Tph1* and TPH2 protein expression in eWAT after 12 weeks of HFD feeding (*n* = 8 for mRNA and 3 for protein per group). (**J**) eWAT 5-HT levels in chow- or HFD-fed mice for 12 weeks (*n* = 12 per group). (**K**) mRNA levels of lipid metabolism–related genes in eWAT after 12 weeks of HFD feeding (*n* = 8 per group). (**L**) Quantification of GAL-3–positive area in eWAT section from HFD-fed mice for 12 weeks (*n* = 5 per group). (**M**) mRNA levels of proinflammatory genes in eWAT after 12 weeks of HFD feeding (*n* = 8 per group). Data are presented as mean ± SEM. For statistical analysis, 2-tailed Student’s *t* test was used. *P < 0.05, **P < 0.01, ***P < 0.001.

5-HT is known to regulate hepatic steatosis by several mechanisms, including directly binding to 5-HTRs on hepatocytes to increase lipogenic gene expression and visceral adipocyte lipolysis in HFD-fed mice (21). H&E staining of liver sections revealed that livers from HFD-KO mice accumulated less lipids, and liver TG levels were lower compared with HFD-Fl mice (Figure 3, A and B). However, no differences were observed between chow-fed mice (Supplemental Figure 2, A and B). Consistent with the reduced hepatic accumulation of TG, HFD-fed mice with adipocyte TPH2 deficiency also had reduced serum ALT and AST levels, indicating protection from hepatic steatosis–induced liver damage (Figure 3, C and D). However, total cholesterol, triglyceride (TG), and nonesterified fatty acids (NEFA) serum levels were not different between HFD-Fl and HFD-KO mice (Supplemental Figure 2, C–E). Quantitative real-time PCR (qPCR) analysis revealed that livers of HFD-KO mice had reduced mRNA expression of several lipogenic genes (Figure 3E). Moreover, mice deficient for adipocyte TPH2 had reduced hepatic expression of genes involved in proinflammatory pathways (Figure 3F). These results indicate that adipocyte-specific TPH2 deletion protected mice from DIO-induced liver damage and hepatic steatosis.

Since we found that HFD-KO mice had reduced eWAT mass compared with HFD-Fl mice, we further investigated the molecular changes induced by adipocyte TPH2 deficiency on the adipose depot. We found that eWAT adipocytes in HFD-KO mice were smaller than those in HFD-Fl (Figure 3, G and H), while Chow-Fl and Chow-KO mice had comparable eWAT adipocyte size (Supplemental Figure 2F). The loss of adipocyte TPH2 reduced the 5-HT production in eWAT of HFD-fed mice, but not in chow-fed mice (Figure 3J and Supplemental Figure 2G). Both TPH2 mRNA and protein levels were decreased in eWAT of HFD-KO mice, without affecting TPH1 expression, leading to a reduction in eWAT 5-HT levels (Figure 3, I and J). Additionally, *Tph2* expression levels were also reduced in both inguinal WAT (iWAT) and mesenteric WAT (mWAT), but *Tph1* mRNA levels in mWAT and ileum were comparable between HFD-Fl and HFD-KO mice (Supplemental Figure 2, H and I), demonstrating that genetic deletion of adipocyte TPH2 did not affect *Tph1* expression in adipose or small bowel depots. Similar to previous studies on the effects of 5-HT on adipocyte gene expression (24), we observed reduced expression of the lipogenic genes *Srebp1c* and *Fasn* and increased expression of the lipolytic genes *Pnpla2* and *Lipe* in HFD-KO mice (Figure 3K). HFD-KO mice had reduced numbers of crown-like structures (CLSs) in eWAT, a canonical histological marker of adipocyte death, and localization of inflammatory macrophages (25, 26) (Figure 3, G and L). Consistent with this observation, in HFD-KO mice, the mRNA levels of proinflammatory genes in eWAT were lower, while *Adipoq* was higher than in HFD-Fl mice (Figure 3M and Supplemental Figure 2J). These findings demonstrate that in DIO mice, adipocyte TPH2–derived 5-HT may promote WAT lipogenesis and inflammation.

### Mice lacking adipocyte TPH2 have increased EE and decreased fecal energy excretion.

**Figure 4:**
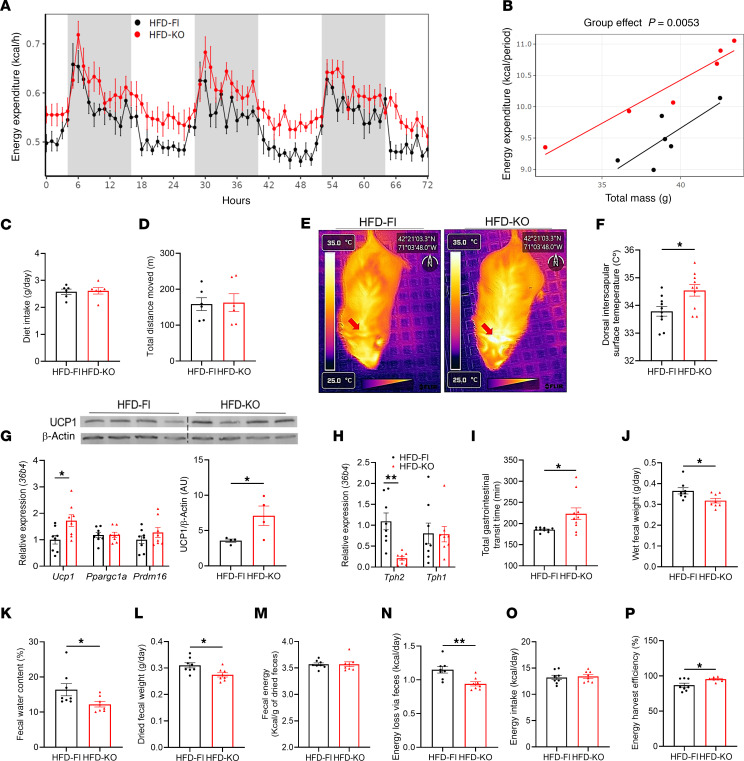
Mice lacking adipocyte TPH2 expression exhibit increased BAT thermogenesis and systemic EE while retaining more energy by reducing fecal energy loss. (**A**) Systemic EE during 72 hours of indirect calorimetry (*n* = 6 per group). (**B**) Regression plot of average EE during 72 hours of measurement. ANCOVA was performed using body weight as a covariate (*n* = 6 per group). (**C** and **D**) Diet intake (**C**) and total distance moved (**D**) during 72 hours of indirect calorimetry measurement (*n* = 6 per group). (**E** and **F**) Representative thermal images of dorsal interscapular area (**E**) and dorsal interscapular surface temperature (**F**) (*n* = 10 per group). (**G**) mRNA levels of *Tph2* and *Tph1* in BAT after 12 weeks of HFD feeding (*n* = 8 per group). (**H**) mRNA levels of thermogenic genes and UCP1 protein expression in BAT after 12 weeks of HFD feeding (*n* = 8 for mRNA and 4 for protein per group). (**I**) Total gastrointestinal transit time after 8 weeks of HFD feeding (*n* = 10 per group). (**J**–**L**) Daily wet feces weight (**J**), water content of feces (**K**), and daily dried fecal weight (**L**) (*n* = 8 per group). (**M**–**P**) Fecal calorie content per gram measured by bomb calorimetry (**M**), daily energy loss via feces (**N**), daily energy intake during the fecal collection period (**O**), and daily level of energy harvest efficiency (**P**) (*n* = 8 per group). Data are presented as mean ± SEM. For statistical analysis, 2-tailed Student’s *t* test was used. *P < 0.05, **P < 0.01.

Although adipocyte TPH2–deficient mice exhibited decreased adipose depot weights and improved glucose homeostasis when challenged with a HFD, a previous study demonstrated that inhibiting adipocyte TPH1 reduced BAT thermogenesis and systemic EE (20), we next investigated the effects of adipocyte TPH2 expression on EE. Surprisingly, HFD-KO mice displayed a higher systemic EE than HFD-Fl mice (Figure 4, A and B). We observed no differences in the daily amount of diet consumed between HFD-Fl and HFD-KO mice nor in the total distance moved, indicating that the group difference in EE is not attributable to differences in physical activity (Figure 4, C and D). To determine if the higher systemic EE in HFD-KO mice is due to increased BAT thermogenesis, we performed infrared thermal imaging of the dorsal interscapular region of mice. We found that the interscapular surface temperature of HFD-KO mice was higher than that of HFD-Fl mice, suggesting increased activation of BAT (Figure 4, E and F). Furthermore, HFD-KO mice had elevated levels of both mRNA and protein for uncoupling protein 1 (UCP1), indicating that the increased BAT thermogenesis is mediated by UCP1 (Figure 4G). Similar to eWAT, BAT *Tph2* mRNA level was decreased in HFD-KO mice, but no changes in *Tph1* expression were observed (Figure 4H).

We next investigated whether the beneficial metabolic effects of adipocyte TPH2 deficiency are present at thermoneutrality. Similar to studies at room temperature, under thermoneutral conditions, HFD-KO and HFD-Fl mice had comparable body composition (Supplemental Figure 3, A–C). In contrast to HFD-KO mice housed at room temperature, there was no difference on the fasting glucose levels between HFD-Fl and HFD-KO mice (Supplemental Figure 3D). The weights of eWAT, iWAT, and liver were similar; however, HFD-KO mice showed reduced BAT mass compared with that of HFD-Fl mice (Supplemental Figure 3, E and F). As expected *Tph2* mRNA levels in both iWAT and BAT from HFD-KO mice were lower than those of HFD-Fl mice under thermoneutral conditions (Supplemental Figure 3, G and H). Interestingly, although *Ucp1* mRNA levels in iWAT were not different between 2 groups, *Ucp1* expression in BAT was increased in HFD-KO mice (Supplemental Figure 3, G–I), suggesting that, even in thermoneutral conditions, adipocyte-derived 5-HT can inhibit BAT metabolism.

Even though the adipocyte-specific deficiency of TPH2 induced BAT thermogenesis and increased EE, paradoxically, the HFD-KO mice still became obese when fed a HFD. As noted above, the amount of diet consumed was not different between HFD-Fl and HFD-KO mice, suggesting energy loss from increased thermogenesis is compensated by other sources. Since 5-HT promotes gut motility, we investigated whether deficiency of adipocyte TPH2 modulated nutrient absorption (27, 28). We hypothesized that the absence of adipocyte TPH2 may enhance energy absorption capacity in the gastrointestinal tract by slowing the rate of nutrient traffic in the intestine, thereby allowing for extended harvesting of calories from the diet. Indeed, HFD-KO mice exhibited significantly delayed total gastrointestinal transit time compared with HFD-Fl mice, suggesting a possible role of adipocyte-derived 5-HT in regulating gut motility (Figure 4I). We subsequently measured fecal output and energy content from mice, and HFD-KO mice produced a reduced amount of feces than HFD-Fl mice (Figure 4J). We also found that both water content and dry mass of fecal samples from HFD-KO mice were reduced (Figure 4, K and L). Bomb calorimetry analysis of fecal samples revealed that energy content per gram of dried feces did not differ between groups, demonstrating that adipocyte TPH2 ablation did not induce caloric malabsorption (Figure 4M). HFD-KO mice exhibited a lower daily caloric loss through feces than HFD-Fl mice, due to their reduced daily fecal output (Figure 4N). We also measured daily energy intake levels during the fecal collection, and there was no difference between the 2 groups (Figure 4O). Our observations align with the hypothesis that HFD-KO mice exhibit increased energy-harvesting efficiency compared with HFD-Fl mice (Figure 4P), which likely contributes to their weight gain under HFD feeding, despite having increased systemic EE. Collectively, these observations suggest that adipocyte TPH2–derived 5-HT not only regulates BAT thermogenesis and systemic EE but also can modulate gut motility and intestinal energy-harvesting efficiency in obese mice.

### Overexpressing adipocyte TPH2 is sufficient to induce obesity without HFD feeding.

**Figure 5:**
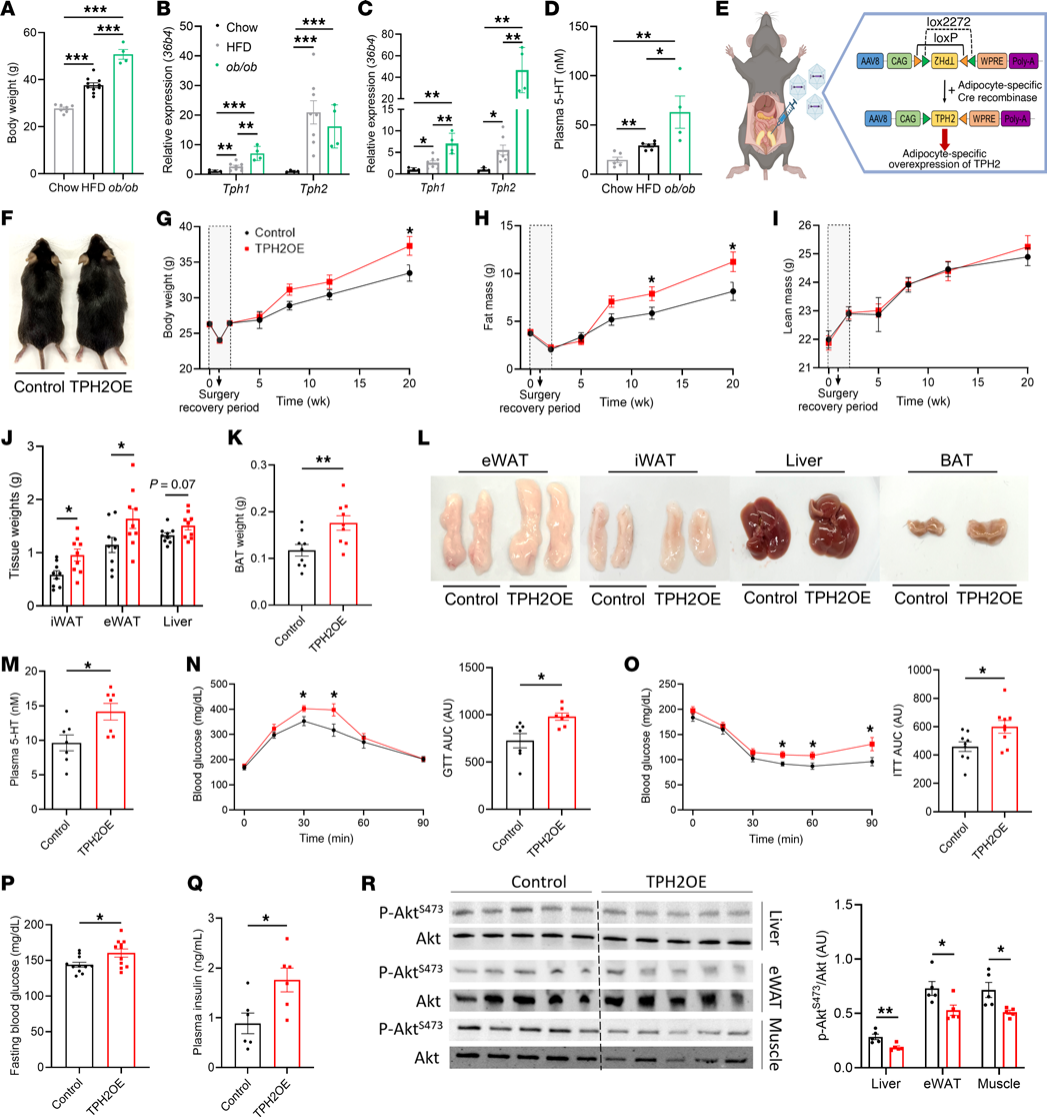
*ob/ob* mice have elevated TPH2 expression in adipocytes, and mice overexpressing eWAT TPH2 develop metabolic dysfunction and have higher levels of circulating 5-HT level in the absence of HFD feeding. (**A**) Body weights of mice after 6 weeks of either chow or HFD feeding (*n* = 7 for chow, 10 for HFD, and 4 for *ob/ob*). (**B**) mRNA levels of *Tph1* and *Tph2* of isolated epididymal white adipocytes in chow- or HFD-fed C57BL6J mice and chow-fed *ob/ob* mice for 6 weeks (*n* = 4 for chow, 8 for HFD, and 4 for *ob/ob*). (**C**) mRNA levels of *Tph1* and *Tph2* of isolated brown adipocytes in chow- or HFD-fed C57BL6J mice and chow-fed *ob/ob* mice for 6 weeks (*n* = 4 for chow, 8 for HFD, and 4 for *ob/ob*). (**D**) Plasma 5-HT levels in *ob/ob* and HFD- and chow-fed mice for 6 weeks (*n* = 6 for chow and HFD; *n* = 4 for *ob/ob*). (**E**) Overview of generating AAV-induced adipocyte-specific TPH2-OE mice. (**F**) Representative photograph of control and TPH2-OE, taken 20 weeks after AAV-TPH2 injection. (**G**–**I**) Time course of body weight (**H**), fat (**I**), and lean mass (**J**) 20 weeks after AAV-TPH2 injection (*n* = 9 per group). (**J**–**L**) Tissue weights (**J** and **K**) and representative photographs of collected tissues (**L**) (*n* = 9 per group). (**M**) Circulating levels of 5-HT from control and TPH2-OE mice, measured 20 weeks after AAV-TPH2 injection (*n* = 6 per group). (**N**) GTT and its AUC, performed 7 weeks after AAV-TPH2 injection (*n* = 7 per group). (**O**) ITT and its AUC, performed 9 weeks after AAV-TPH2 injection (*n* = 8 per group). (**P** and **Q**) Fasting blood glucose (*n* = 9 per group) and plasma insulin levels (*n* = 6 per group), measured 20 weeks after AAV-TPH2 injection. (**R**) Relative levels of phospho-Akt (Ser473) to total Akt in liver, eWAT, and gastrocnemius muscle of control and TPH2-OE mice, 8 weeks after AAV-TPH2 injection with insulin stimulation (15 minutes, *n* = 5 per group). Data are presented as mean ± SEM. For statistical analysis, 1-way ANOVA with Tukey’s multiple-comparison test (**A**–**D**), 2-way ANOVA with Dunnett’s test (**G**–**I**), or 2-tailed Student’s *t* test (**J**, **K**, and **M**–**R**) was used. *P < 0.05, **P < 0.01, ***P < 0.001.

To further investigate whether a HFD itself or obesity-associated changes result in dramatic upregulation in adipocyte *Tph2* expression, we analyzed *Tph2* mRNA levels in age-matched chow-fed C57BL/6J and *ob/ob* mice or C57BL/6J mice fed a HFD for 6 weeks. HFD-fed C57BL6 and chow-fed *ob/ob* mice became substantially obese compared with control mice (Figure 5A). In isolated eWAT adipocytes of HFD-fed mice and *ob/ob* mice, we observed a significant increase in the expression of both *Tph1* and *Tph2* compared with chow-fed C57BL/6J mice (Figure 5B). After just 2 weeks on a HFD, Tph2 mRNA levels were greater in eWAT compared with chow-fed mice. (Supplemental Figure 4A). Similarly, the mRNA levels of both *Tph1* and *Tph2* were upregulated in isolated brown adipocytes of HFD-fed mice, and we observed an even greater increase in *ob/ob* mice (Figure 5C). Plasma 5-HT levels were also increased in HFD C57BL/6J mice, and even higher levels were found in *ob/ob* mice (Figure 5D). These findings suggest that obesity-induced upregulation of TPH2 occurs independently of HFD feeding, indicating that pathways associated with obesity, not the type of diet, are responsible for the increased adipocyte TPH2 upregulation.

Although HFD-KO mice exhibited phenotypes distinct from adipocyte TPH1-KO mice fed a HFD, it remains unclear whether these phenotypes specifically result from adipocyte TPH2 deficiency or are influenced by a combination of other factors, such as HFD feeding and variations in energy-harvesting efficiency. Because feeding mice a HFD results in DIO and multiple changes in metabolism and inflammation (29–31), to delineate the specific metabolic effects of adipocyte TPH2 expression, we overexpressed TPH2 exclusively in eWAT adipocytes of C57BL/6J mice without HFD feeding. We utilized an adeno-associated virus (AAV) flip-excision conditional Cre-Switch vector carrying mouse TPH2 cDNA in antisense orientation (AAV-TPH2), which, in the presence of cellular Cre recombinase, results in the sense orientation of TPH2 cDNA, leading to the overexpression of TPH2, specifically in eWAT adipocytes (Supplemental Figure 4B). For these studies, we performed laparotomies to directly inject AAV-TPH2 into eWAT of mice with or without the Adipoq-Cre recombinase (control and TPH2-OE, respectively; Figure 5E). *Tph2* was overexpressed only in eWAT adipocytes of mice carrying the Adipoq-Cre recombinase (Supplemental Figure 4C). TPH2-OE mice on a chow diet gradually gained more body weight and fat mass compared with control mice, with no differences observed in lean mass (Figure 5, F–I). Notably, weights of eWAT, iWAT, and BAT depots were significantly increased in TPH2-OE mice, and liver weights showed a higher trend (*P* = 0.07; Figure 5, J–L). Overexpressing TPH2 only in eWAT adipocytes alone was sufficient to elevate circulating levels of 5-HT, thereby confirming the potential contribution of adipocyte TPH2 to the peripheral 5-HT circulating pool (Figure 5M). TPH2-OE mice exhibited glucose intolerance and insulin resistance relative to control mice (Figure 5, N and O). TPH2-OE mice also had elevated fasting blood glucose and plasma insulin concentrations compared with control mice (Figure 5, P and Q). To further evaluate the impact of adipocyte TPH2 overexpression on insulin sensitivity, we investigated the effects of insulin stimulation on phospho-Akt/total Akt ratio. Consistent with the insulin tolerance test (ITT) and glucose tolerance test (GTT) results, TPH2-OE mice exhibited a reduced phospho-Akt ratio in metabolic tissues, such as liver, eWAT, and muscle compared with control mice (Figure 5R). Overall, TPH2 overexpression in eWAT potentiates weight gain and impedes insulin signaling in metabolic tissues, disrupting systemic glucose homeostasis.

### Overexpression of eWAT adipocyte TPH2 induces adipocyte dysfunction and hepatic steatosis without HFD feeding.

**Figure 6:**
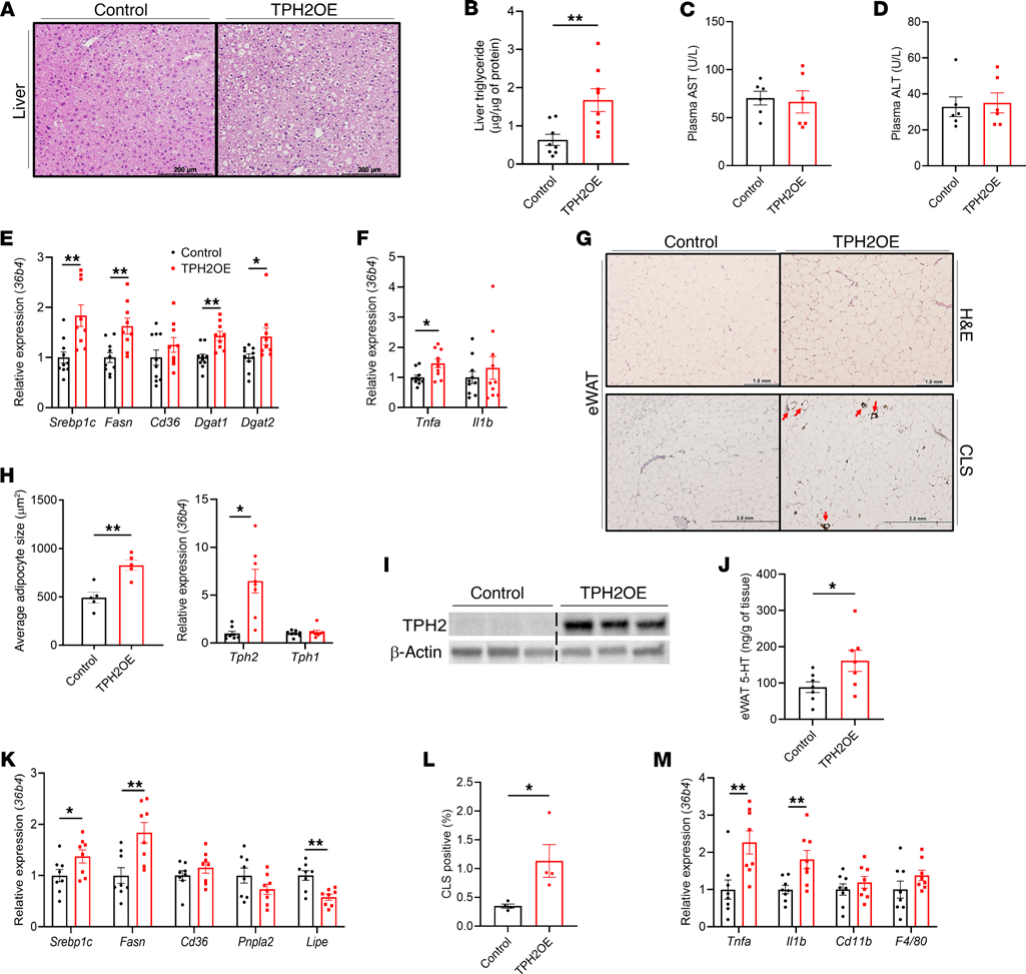
Adipocyte-specific TPH2 overexpression induces hepatic steatosis and adipocyte dysfunction without HFD feeding. (**A**) Representative images of H&E-stained liver, 20 weeks after AAV-TPH2 injection (*n* = 5 per group). Scale bars: 200 μm. (**B**) TG levels in liver of mice, 20 weeks after AAV-TPH2 injection (*n* = 8 per group). (**C** and **D**) Plasma levels of ALT (**C**) and AST (**D**) (*n* = 6 per group). (**E** and **F**) mRNA levels of lipid metabolism–related genes (**E**) and inflammatory genes (**F**) in liver, 20 weeks after AAV-TPH2 injection (*n* = 10 per group). (**G**) Representative images of H&E staining and GAL-3 (MAC-2) immunohistochemical staining of eWAT showing CLS formation (indicated by red arrows), 20 weeks after AAV-TPH2 injection (*n* = 5 for H&E 4 for CLS per group). Scale bars: 1 mm (top), 2 mm (bottom). (**H**) Average adipocyte size of mice, 20 weeks after AAV-TPH2 injection (*n* = 5). (**I**) mRNA levels of *Tph2* and *Tph1* and TPH2 protein expression in eWAT, 20 weeks after AAV-TPH2 injection (*n* = 8 for mRNA and 3 for protein per group). (**J**) eWAT 5-HT levels in control and TPH2-OE mice, 20 weeks after AAV injection (*n* = 7 per group). (**K**) mRNA levels of lipid metabolism–related genes in eWAT, 20 weeks after AAV-TPH2 injection (*n* = 8 per group). (**L**) Quantification of GAL-3 positive area in eWAT section, 20 weeks after AAV-TPH2 injection (*n* = 4 per group). (**M**) mRNA levels of proinflammatory genes in eWAT, 20 weeks after AAV-TPH2 injection (*n* = 8 per group). Data are presented as mean ± SEM. For statistical analysis, 2-tailed Student’s *t* test was used. *P < 0.05, **P < 0.01.

We next investigated the molecular changes in TPH2-OE mice to elucidate the mechanism of how eWAT adipocyte-specific overexpression of TPH2 contributes to metabolic dysfunction. Histological and molecular assessment of the liver revealed that TPH2 overexpression increased hepatic lipid accumulation (Figure 6, A and B). However, in the absence of HFD-induced changes, this was not sufficient to induce significant changes in plasma levels of AST, ALT, and serum lipids such as total cholesterol, TG, and NEFA (Figure 6, C and D, and Supplemental Figure 4, D–F). Consistent with increased hepatic lipid accumulation in TPH2-OE mice, mRNA levels of genes involved in lipogenesis and the proinflammatory cytokine gene *Tnfa* were increased (Figure 6, E and F). Altogether, our results indicate that overexpression of TPH2 in eWAT adipocytes promotes hepatic lipid accumulation without HFD challenge in mice.

In accordance with increased eWAT mass in TPH2-OE mice, H&E staining of eWAT revealed that the average size of eWAT adipocytes in TPH2-OE mice was larger compared with that of control mice (Figure 6, G and H). We further assessed the molecular changes with TPH2 overexpression and found that AAV-TPH2 increased both TPH2 mRNA and protein expression in eWAT of TPH2-OE mice but had no effect on *Tph1* expression (Figure 6I). Of note, 5-HT levels in eWAT were elevated, demonstrating the contribution of adipocyte TPH2 in adipocyte 5-HT production (Figure 6J). eWAT of TPH2-OE mice exhibited increased expression of lipogenic genes and reduced mRNA expression of the lipolytic gene *Lipe* (Figure 6K). Furthermore, we observed that TPH2 overexpression provoked formation of CLSs in eWAT and increased expression of proinflammatory markers (Figure 6, L and M). In contrast to the adipocyte TPH2 knockout model, the mRNA level of *Adipoq* was not different in eWAT between control and TPH2-OE mice (Supplemental Figure 4G). A previous study demonstrated that 5-HT induces lipogenesis in white adipocytes via 5-HTR2, while thermogenesis in brown adipocytes was suppressed via 5-HTR3 (20). To investigate whether adipocyte TPH2–derived 5-HT also regulates adipocyte metabolism via the same pathways, we overexpressed TPH2 in differentiated primary white adipocytes using TPH2-AAV and Cre recombinase expressing AAV (Cre-AAV). TPH2-AAV only upregulated *Tph2* mRNA levels in cells with Cre-AAV, and treating cells with ketanserin (KET), a 5-HTR2 antagonist, did not alter *Tph2* and *Tph1* gene expression levels (Supplemental Figure 5, A and B). As expected, TPH2 overexpression led to increased production and secretion of 5-HT from adipocytes and increased 5-HT levels in media (Supplemental Figure 5C). Overexpression of TPH2 in primary white adipocytes upregulated expression of lipogenic genes, such as *Fasn*, *Dgat1*, and *Dgat2*, compared with vehicle-treated cells, and this upregulation diminished with KET treatment, indicating that adipocyte TPH2–derived 5-HT can autonomously regulate lipogenesis via 5-HTR2 (Supplemental Figure 5, D–F).

Collectively, these observations further substantiate the role of adipocyte TPH2 in the development of metabolic dysfunction, indicating that 5-HT originating exclusively from adipocyte TPH2 is sufficient to induce hepatic lipid accumulation and adipocyte dysfunction even in the absence of obesogenic insults.

### Overexpression of TPH2 in eWAT adipocytes inhibits BAT thermogenesis and decreases systemic EE.

**Figure 7:**
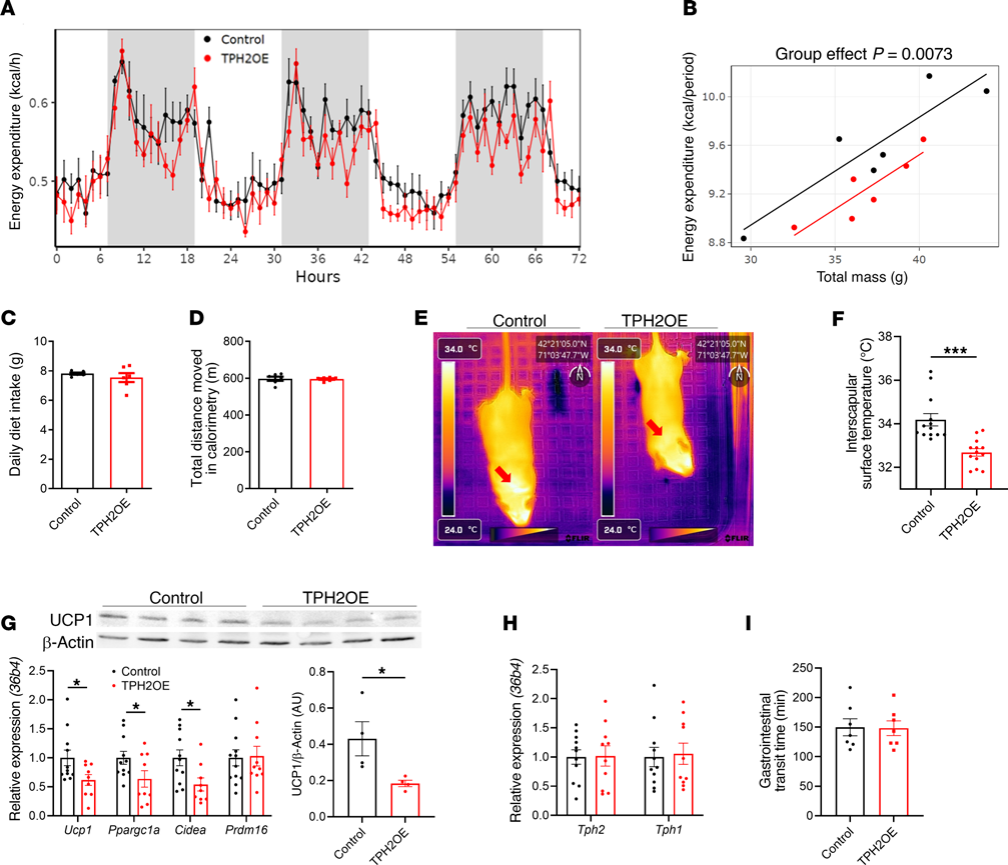
TPH2 overexpression in epididymal adipocytes decreases BAT thermogenesis and systemic EE. (**A**) Systemic EE during 72 hours of indirect calorimetry (*n* = 6 per group). (**B**) Regression plot of average EE during 72 hours of measurement. ANCOVA was performed using body weight as a covariate (*n* = 6 per group). (**C** and **D**) Diet intake (**C**) and total distance moved (**D**) during 72 hours of indirect calorimetry measurement (*n* = 6 per group). (**E** and **F**) Representative thermal images of dorsal interscapular area (**E**) and dorsal interscapular surface temperature (**F**) (*n* = 13 per group). (**G**) mRNA levels of *Tph2* and *Tph1* in BAT, 20 weeks after AAV-TPH2 injection (*n* = 10 per group). (**H**) mRNA levels of thermogenic genes and UCP1 protein expression in BAT, 20 weeks after AAV-TPH2 injection (*n* = 10 for mRNA and 4 for protein per group). (**I**) Total gastrointestinal transit time, 8 weeks after AAV-TPH2 injection (*n* = 10 per group). Data are presented as mean ± SEM. For statistical analysis, 2-tailed Student’s *t* test was used. *P < 0.05, **P < 0.01, ***P < 0.001.

The overexpression of TPH2 in eWAT adipocytes not only perturbed adipocyte metabolism but also detrimentally altered the systemic physiology of mice. We observed that systemic EE in TPH2-OE was reduced compared with that in control mice (Figure 7, A and B). No differences in daily diet intake or physical activity were observed between the 2 groups (Figure 7, C and D). To determine the potential BAT dysfunction in TPH2-OE mice, we performed infrared thermal imaging on the dorsal interscapular region of mice. TPH2-OE mice demonstrated reduced surface temperature compared with control mice (Figure 7, E and F). Also, in BAT of TPH2-OE mice, mRNA levels of thermogenic genes, such as *Ucp1* and *Ppargc1a*, were reduced, and UCP1 expression in BAT was also decreased, highlighting the impact of eWAT adipocyte–derived 5-HT on regulating BAT thermogenesis (Figure 7G). Consistent with the specificity of expression in eWAT of TPH2-OE mice, no differences in *Tph1* or *Tph2* expression in BAT were observed between the 2 groups of mice (Figure 7H). Additionally, contrary to what was observed in the adipocyte TPH2 knockout model, the total gut transit time remained unaltered (Figure 7I), indicating no alterations in intestinal energy harvesting. These data suggest that the increase in circulating 5-HT levels induced by overexpression of TPH2 in eWAT reduced BAT activity and thermogenesis; however, it was insufficient to influence gut motility and gastrointestinal transit in chow-fed mice.

We next investigated whether thermoneutrality blunts the metabolic effects of eWAT TPH2–derived 5-HT. In contrast to our observations in TPH2-OE mice housed at room temperature, TPH2-OE mice on a chow diet exhibited similar body composition and fasting glucose levels after 8 weeks of thermoneutral housing (Supplemental Figure 6, A and D). This suggests that the suppression of BAT thermogenesis under thermoneutral conditions mitigated the effects of adipocyte TPH2–derived 5-HT. To further investigate the mechanisms by which TPH2 regulates brown adipocyte metabolism, we overexpressed TPH2 in primary brown adipocytes. In differentiated brown adipocytes, TPH2-AAV also increased *Tph2* expression, not *Tph1,* while CL316243 (CL; a β3-adrenoceptor agonist) or ondansetron (ODS; a 5-HTR3 antagonist) treatment did not affect*Tph2* and *Tph1* gene expression levels (Supplemental Figure 5, G and H). Consistent with findings from white adipocyte TPH2 overexpression, upregulation of TPH2 in brown adipocytes also increased 5-HT levels in media compared with that of vehicle-treated cells (Supplemental Figure 5I). TPH2-derived 5-HT reduced Ucp1 mRNA levels and inhibited CL-induced increases in *Ucp1* expression. However, ODS treatment completely abrogated the effects of 5-HT on suppressing *Ucp1* expression and restored *Ucp1* levels in TPH2-AAV–treated brown adipocytes, both in the presence and absence of CL stimulation. (Supplemental Figure 5J). Additionally, TPH2 overexpression in primary brown adipocytes resulted in decreased free glycerol levels in the media compared with control cells, and this reduction was reversed with ODS treatment (Supplemental Figure 5K). These results demonstrate the cell-autonomous effects of 5-HT in regulating thermogenesis in brown adipocytes.

While TPH2-OE mice exhibited reduced BAT thermogenesis, they also displayed increased iWAT mass, which might be secondary to increased circulating levels of 5-HT. To determine the possible mechanisms for this observation, we analyzed molecular changes in iWAT of TPH2-OE mice. Interestingly, eWAT adipocyte TPH2 overexpression greatly increased inguinal adipocyte size without affecting *Tph1* or *Tph2* expression (Supplemental Figure 6, E and G). In iWAT, *Ucp1* and *Ppargc1a* mRNA levels were not different between the 2 groups (Supplemental Figure 6H), suggesting that AAV-TPH2 did not alter iWAT thermogenesis. Additionally, qPCR analysis revealed that TPH2-OE mice had increased iWAT expression of genes associated with lipogenesis and decreased levels of the lipolytic gene *Pnpla2*, relative to their respective control (Supplemental Figure 6I). We found no changes in mRNA levels of genes involved in proinflammatory pathways (Supplemental Figure 6J). These data suggest that increased circulating levels of 5-HT in TPH2-OE mice upregulated lipogenic gene expression in iWAT, resulting in larger adipocytes.

Taken together, these results underscore that selectively augmenting 5-HT production by TPH2 exclusively in eWAT suppresses BAT thermogenesis and EE, while inducing hepatic steatosis and iWAT lipogenesis, leading to the onset of metabolic abnormalities independent of excess caloric intake.

### Insulin regulates adipocyte TPH2 expression via Akt/mTORC1/SREBP1 pathway.

**Figure 8:**
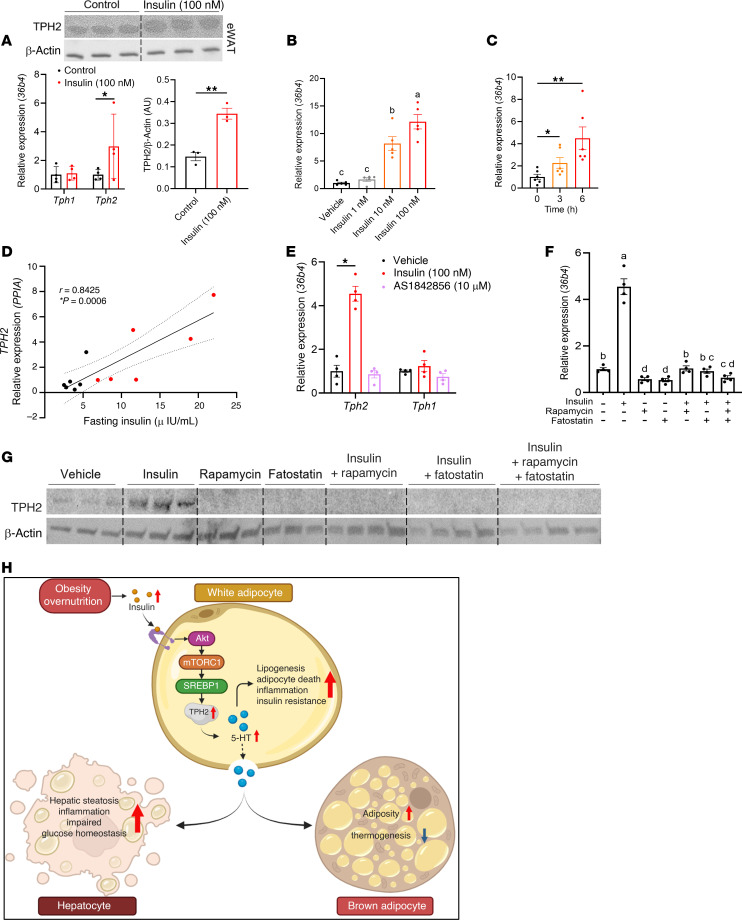
Insulin signaling promotes adipocyte TPH2 expression via the Akt/mTORC1/SREBP1 pathway. (**A**) mRNA levels of *Tph1*, *Tph2*, and TPH2 protein expression in explants from eWAT after 6 hours of insulin (100 nM) stimulation (*n* = 4 for mRNA and 3 for protein per group). (**B**) mRNA levels of *Tph2* in differentiated adipocytes from iWAT preadipocytes after 8 hours of different concentrations of insulin treatment (*n* = 5 per group). (**C**) mRNA levels of *Tph2* in differentiated adipocytes from iWAT preadipocytes after 0, 3, and 6 hours of insulin (100 nM) treatment (*n* = 5 per group). (**D**) Correlation between *TPH2* expression in human subcutaneous fat and fasting insulin levels of lean and obese individuals (*n* = 6 per group), Pearson’s *r* correlation coefficient with corresponding *P* values. (**E**) mRNA levels of *Tph2* and *Tph1* in differentiated adipocytes from iWAT preadipocytes after 6 hours of insulin or AS1842586 (10 μM) treatment (*n* = 4 per group). **(F**) mRNA levels of *Tph2* in differentiated adipocytes from iWAT preadipocytes after 6 hours of insulin, rapamycin (25 μM), and/or fatostatin (20 μM) treatment (*n* = 3~4 per group). (**G**) TPH2 protein expression in differentiated adipocytes from iWAT after 6 hours of insulin, rapamycin (25 μM), and/or fatostatin (20 μM) treatment (*n* = 4 per group). (**H**) Graphical summary of how obesity promotes TPH2 expression and the role of TPH2 in developing obesity-induced metabolic dysfunction. Data are presented as mean ± SEM. For statistical analysis, 2-tailed Student’s *t* test (**A**), 1-way ANOVA with Tukey’s multiple-comparison test (**B**–**D**), or Welch and Brown-Forsythe ANOVA (**E**) was used. *P < 0.05, **P < 0.01; lowercase letters indicate statistical difference between treatments, *P* < 0.05.

Both HFD-fed mice and chow-fed *ob/ob* mice had striking increases in *Tph2* mRNA levels, suggesting obesity-associated pathways, not specific diets, are responsible for this upregulation. Because obesity per se is associated with hyperinsulinemia and insulin plays a pivotal role as a regulator of crucial transcription factors (32), we hypothesized that obesity-induced hyperinsulinemia can upregulate adipocyte TPH2 expression. To investigate the potential role of insulin in regulating adipocyte TPH2 expression, we incubated eWAT explants from C57BL/6J mice with insulin and observed robust elevation of both TPH2 mRNA and protein levels without altering expression of *Tph1* (Figure 8A). Furthermore, we differentiated preadipocytes from the stromal vascular fraction (SVF) of iWAT from C57BL/6J mice into mature adipocytes and incubated the cells with differing durations and concentrations of insulin. *Tph2* mRNA levels increased in a time- and dose-dependent manner with insulin stimulation (Figure 8, B and C), indicating the direct relationship between insulin and TPH2 expression. To further investigate the physiological relevance of the insulin-induced upregulation of adipocyte TPH2 expression, we evaluated the relationship between *TPH2* mRNA levels in human subcutaneous adipose tissue and fasting insulin concentrations. Remarkably, *TPH2* mRNA levels showed a strong positive correlation with fasting plasma insulin levels in human subjects (Figure 8D). To assess the effect of prandial circulating insulin levels on adipocyte *Tph2* expression in vivo, we examined *Tph2* expression in adipose depots of chow-fed C57BL6/J mice subjected to overnight fasting and refeeding, conditions in which insulin concentrations decrease and then increase, respectively. We observed that adipocyte *Tph2* mRNA levels decreased with fasting and increased with refeeding in iWAT, eWAT, and BAT (Supplemental Figure 7, A and C). In summary, these data suggest that obesity-induced hyperinsulinemia increases expression of adipose tissue TPH2 in both obese mice and humans, which might contribute to increased circulating 5-HT and potentially the progression of obesity-associated metabolic complications.

To determine the metabolic pathways underlying the insulin-induced upregulation of adipocyte *Tph2* and specifically which intracellular insulin signaling pathway regulates *Tph2* transcription, we first investigated whether forkhead box protein 01 (FOXO1) inhibition mimics the effect of insulin on *Tph2* upregulation (33). A selective FOXO1 inhibitor, AS1842856, treatment did not alter *Tph2* or *Tph1* expression in differentiated adipocytes, demonstrating that *Tph2* regulation is not mediated by the Akt/FOXO1 pathway (Figure 8E). Another important downstream target of canonical insulin signaling pathway is mTOR (34). Since we observed that adipocyte TPH2 expression increases expression of lipogenic genes, we hypothesized that SREBP1, an important transcription factor crucial for lipid biosynthesis and regulated by mechanistic target of rapamycin complex 1 (mTORC1), might control TPH2 expression along with other lipid metabolism enzymes (35). To test this hypothesis, we inhibited mTORC1 or SREBP1 by treating adipocytes with either a selective mTOR1C inhibitor, rapamycin, or fatostatin, which inhibits SREBP1 activation (36, 37). Remarkably, both rapamycin and fatostatin treatment downregulated *Tph2* mRNA expression in the absence of additional exogenous insulin and also suppressed insulin-induced increases in *Tph2* expression (Figure 8, F and G). Furthermore, inhibition of both mTORC1 and SREBP1 completely diminished insulin-stimulated upregulation of TPH2 (Figure 8, F and G). These findings highlight the regulation of adipocyte TPH2 via the Akt/mTORC1/SREBP1 pathway, offering insights into the underlying mechanism of the dramatic increase of adipocyte TPH2 in DIO and suggesting a possible therapeutic pathway for ameliorating metabolic dysregulation.

## Discussion

Adipocytes secrete various adipokines and bioactive substances that orchestrate both local and systemic metabolism to maintain optimal metabolic homeostasis (35). An important consequence of obesity is that the disrupted pattern of adipokines and metabolites from adipocytes contributes to alterations in systemic metabolism (38). One of the DIO-induced changes in adipose tissue is increased biosynthesis of 5-HT by adipocytes (19). Previously, obesity-associated increases in adipocyte TPH1 and the corresponding elevation in local adipose tissue 5-HT have been shown to alter the metabolism of both white and brown adipocytes (19, 20). In the current study, we demonstrate that in the obese state, adipocyte TPH2, an isoform mainly expressed in neural cells, is highly expressed and contributes to both local adipose and circulating levels of 5-HT, which has significant effects on local adipose tissue and distal tissue physiology.

Initially, both peripheral and central 5-HT were believed to be synthesized by TPH1 (39). However, in 2003, Walther et al. discovered the existence of TPH2, a second isoform of TPH, and also found that TPH1 is not expressed in the brain (40). Because 5-HT cannot cross the blood–brain barrier, the CNS and peripheral 5-HT pools are separately maintained and regulated, and TPH2 was considered to exclusively regulate biosynthesis of neuronal 5-HT (41). However, in this study, we found that both *ob/ob* and HFD-fed obese mice have increased adipocyte TPH2 expression and plasma 5-HT levels, indicating the association between adipocyte TPH2 and peripheral 5-HT production. In chow-fed mice, we demonstrated that TPH2 overexpression specifically in eWAT adipocytes increased both local adipose tissue and circulating levels of 5-HT. The increased circulating 5-HT was sufficient to reduce BAT thermogenesis and systemic EE via 5-HTR activation, while promoting weight gain, iWAT lipogenesis, hepatic steatosis, and systemic insulin resistance.

Interestingly, even though HFD-fed mice with adipocyte-specific TPH2 deficiency demonstrated increased BAT thermogenesis and systemic EE, they were not protected against diet-induced weight gain. In the gastrointestinal system, 5-HT can bind to 5-HTR4 to increase gut motility by inducing peristalsis (42). Genetic ablation of gut enterochromaffin cell–specific TPH1 resulted in inhibition of gut peristalsis and slower gastric emptying in mice (27). Our studies indicated that HFD-KO mice exhibited reduced gut motility, which was associated with decreased fecal energy loss. This finding suggests that enhanced intestinal harvesting of dietary calories is at least one factor contributing to the observed similarities in body composition between HFD-Fl and HFD-KO mice. While crosstalk between adipose tissues and other metabolic tissues is well recognized, there are very few studies demonstrating direct crosstalk between factors generated by adipocytes that regulate intestinal nutrient absorption. A recent study discovered the role of adipocyte-stored iron in regulating intestinal lipid absorption and the progression of DIO, underscoring the importance of the adipocyte-gut axis on systemic metabolism (43). However, contrary to HFD-fed adipocyte-specific TPH2 knockout mice, we did not observe any effects on gut motility in mice with eWAT TPH2 overexpression compared with control mice. One possible explanation for this observation would be that, in mice fed a HFD, adipocyte TPH2 expression is increased in multiple adipose depots including mWAT (44). mWAT is located adjacent and in direct contact with the intestinal serosa and is important for maintaining optimal intestinal function and metabolic homeostasis (45, 46). Considering the substantial contribution from adipocyte TPH2 to peripheral 5-HT biosynthesis, mWAT TPH2–produced 5-HT might affect the gastrointestinal system, resulting in a small, but substantial, alteration of gut motility and increased intestinal harvesting of calories from the HFD. These findings underscore the substantial role of adipocytes in governing whole-body energy balance, warranting further investigation of the role of adipocyte TPH2 for regulating intestinal function and the possible interaction with gut microbiome profile in intestinal energy metabolism.

An important consequence of obesity is hyperinsulinemia (47). Insulin is a key anabolic hormone well known for its role in facilitating postprandial cellular glucose uptake and stimulating various metabolic pathways, including those involved in protein and lipid metabolism (48). Many downstream metabolic pathways regulated by insulin are mediated by the PI3K/Akt pathway, and one of the major targets of the PI3K/Akt pathway is mTOR (49). The mTOR pathway is composed of 2 distinct complexes, mTORC1 and mTORC2, which integrate nutrient and hormonal signals to control cell growth and proliferation (50). With insulin-stimulated Akt activation, mTORC1 induces both transcription and activation of SREBP1, a transcription factor important for regulating genes associated with adipogenesis and lipid homeostasis (51). In our studies, we demonstrated that both DIO and *ob/ob* mice have elevated fasting serum insulin levels in serum and increased adipocyte TPH2 expression. The coordinated changes in adipose TPH2 expression observed in our studies on fasted and refed mice, with corresponding low and high insulin concentrations, are consistent with a direct effect of insulin on the regulation of this pathway. Consistent with a possible role for insulin in adipocyte TPH2 expression, we found that incubating eWAT explants and primary adipocytes with insulin resulted in a marked upregulation of TPH2 expression. Notably, the pharmacological inhibition of mTORC1 or SREBP1 diminished insulin-induced upregulation of adipocyte TPH2, suggesting the potential role of insulin in the transcriptional regulation of adipocyte TPH2 expression. Additional studies are needed to elucidate why insulin selectively regulates TPH2, rather than TPH1, in adipocytes, as well as to determine the specific transcriptional mechanisms through which SREBP1 modulates adipocyte TPH2 expression.

In the current study, we demonstrated that obese humans with hyperinsulinemia have increased adipose tissue TPH2 expression. *TPH2* mRNA levels of subcutaneous adipose tissue showed a positive linear correlation with fasting plasma AST and plasma insulin levels in humans, suggesting that obesity-induced hyperinsulinemia increases TPH2 expression in human adipose tissues. Of potential relevance to our observation, previous studies reported that circulating 5-HT levels are positively correlated with body mass index and hemoglobin A1c in obese humans (52). Also of related interest, in a separate study of overweight individuals with metabolic syndrome, urinary excretion of 5-hydroxyindoleacetic acid, the primary metabolite of 5-HT, was increased (53). In mice, peripheral 5-HT alters both adipose and hepatic metabolism to induce insulin resistance and hepatic steatosis, while inhibiting UCP1 expression and uncoupled respiration in BAT, resulting in decreased systemic EE (19). Although, in human adults, it is unclear whether BAT significantly contributes to systemic EE, BAT activation may still lead to metabolic benefits (54). Previously, by using ^18^F-fluorodeoxyglucose positron emission tomography, retrospective studies demonstrated that adult humans with BAT had lower prevalences of cardiometabolic diseases, including hepatic steatosis and T2DM, than individuals without BAT (55–57). Interestingly, incubating differentiated human brown adipocytes with 5-HT was found to inhibit noradrenaline-stimulated uncoupled respiration, demonstrating a possible role of 5-HT in regulating BAT thermogenic capacity in humans (58). Taken together, future studies are needed to further investigate the possible interactions and clinical relevance between hyperinsulinemia-induced adipocyte TPH2 expression, circulating levels of 5-HT, and their effects on systemic metabolism and metabolic disorders.

The present data highlight how obesity regulates the expression of adipocyte TPH2 and its role in regulating both white and brown adipocytes and systemic energy metabolism, providing a cellular signaling pathway that links obesity-associated hyperinsulinemia to increased adipocyte TPH2 expression (Figure 8H). In conclusion, our study indicates that TPH2, the predominant TPH isoform in the CNS, is dramatically upregulated in adipocytes by obesity-induced hyperinsulinemia altering systemic metabolism, and adipocyte-specific inhibition of TPH2 could be a promising therapeutic intervention for DIO and its metabolic complications.

## Methods

### Sex as a biological variant.

All experimental animals used in this study were male because female C57BL/6J mice are less susceptible to develop HFD-induced obesity and insulin resistance. It is unknown whether the findings are relevant for female mice. For human data, both male (*n* = 5) and female (*n* = 7) participants were included.

### Experimental animals.

Adipocyte-specific TPH2-deficient mice were generated by mating mice possessing *loxP* sites flanking exon 5 of the *Tph2* (TPH2^loxP/loxP^, B6;129S7-Tph2tm1Zfc/J) and Adipoq-Cre transgene–expressing mice [B6.FVB-Tg(Adipoq-cre)1Evdr/J] purchased from The Jackson Laboratory. Heterozygous floxed mice with hemizygous Adipoq-Cre were mated again with *Tph2* loxP heterozygotes to generate TPH2^loxP/loxP^ mice with the hemizygous Adipoq-Cre gene. Six-week-old *ob/ob* mice (B6.Cg-Lepob/J) were purchased from The Jackson Laboratory and housed under the same environment with other mice. Mice had unrestricted access to either chow diet (2916; Teklad) or a HFD (D12492; Research Diets) according to their groups during the whole experimental period. Mice were housed at either room temperature (23°C) or thermoneutrality (30°C) in the Comparative Biology Unit at the Jean Mayer USDA Human Nutrition Research Center on Aging. Prior to specimen collection, mice were fasted for 6 hours. Under continuous isoflurane anesthesia, cardiac puncture was carried out to collect blood. Collected blood was transferred to both EDTA-coated tubes and noncoated 2 mL tubes for plasma and serum isolation, respectively. All collected tissues were weighed for their mass and immediately snap-frozen in liquid nitrogen.

### Generation of TPH2-overexpressing AAV.

The plasmid construct pAAV-CAG-DIO-Tph2 (antisense orientation)-WPRE was acquired from Vector Biolabs (Supplemental Figure 3B). The plasmid vector was amplified using a chemically competent *E*. *coli* system (OneShot Stbl3; Invitrogen). Briefly, the construct was incubated on ice with OneShot *E*. *coli*. After short cold exposure, cells were heat-shocked (42°C) and then incubated at 37°C with gentle shaking. The transformant was spread on a prewarmed Luria broth agar plate containing ampicillin (100 μg/mL). After overnight incubation, amplified plasmid was purified using the column method (Plasmid Maxi kit; QIAGEN). To validate the presence of *Tph2* gene in purified plasmid, a restriction enzyme–based diagnostic test was performed. Verified plasmid was sent to Boston Children’s Hospital Viral Core and packaged into AAV8 serotype, and the final titer of AAV-TPH2 was 3.0 × 10^13^ GC/mL.

### AAV injection.

To directly inject AAV into eWAT, laparotomies were performed. Mice with or without Adipoq-Cre expression were anesthetized with isoflurane. Each epididymal adipose depot was carefully brought out, and 20 μL of AAV (titer: 3.0 × 10^12^ GC/mL) was injected at 5 different points throughout each epididymal fat pad (2 μL per point of injection). The peritoneal cavity was closed with absorbable sutures, and wound clips were used to close the outer skin wound. For postoperative pain, mice were given slow-releasing buprenorphine (0.1 mg/kg) by intraperitoneal injection before the procedure. Mice were individually caged and closely monitored for any signs of infection or distress. Wound clips were removed from mice after 10 days from the procedure.

### Metabolic phenotyping.

The mouse body composition (lean, fat, and body mass) was analyzed using MRI (EchoMRI-700; EchoMRI) every 3 weeks. Diet intake was determined daily by measuring the weight of remaining diet pellets on feeders at 10 am. Systemic EE was analyzed using an indirect calorimetry system (Promethion BX1; Sable Systems). The first 24 hours of collected data were excluded from analysis for acclimation purposes. Collected metabolic data were analyzed by CalR as previously described (59). For GTTs and ITTs, mice were transferred to new cages without food pellets as soon as the light cycle started and fasted for 6 hours. After fasting, a tail bit was snipped to measure baseline blood glucose (Autocode; Prodigy) before sacrificing mice. The glucose solution (d-glucose, Sigma, diluted with sterile PBS) or insulin solution (Humulin R U-100, Eli Lily, diluted with sterile PBS) was intraperitoneally injected, and blood glucose level was measured. AUC was calculated based on average glucose level at each time point. For fasting and refeeding experiments, mice were fasted overnight for approximately 16 hours, refed with chow diet for 2 hours, and then sacrificed for specimen collection.

### Infrared imaging of dorsal interscapular area.

The temperature of the dorsal interscapular region was measured with an infrared thermal camera (FLIR One Pro LT iOS). Within 2 hours after the light cycle began, mice were carefully brought from the cages (without directly touching them), and thermal images of mice were taken without shaving their back hair for 3 days. The average temperature of the interscapular region was analyzed with the FLIR thermal studio suite.

### Plasma and serum profile analysis.

Serum total cholesterol, TG, ALT, and AST were analyzed using diagnostic reagents on a chemistry analyzer (Beckman Coulter AU480). NEFA was analyzed using the Wako NEFA-HR test kit and analyzed on the AU480 chemistry analyzer. Plasma insulin was measured by ELISA (Ultra-Sensitive Mouse Insulin ELISA; Crystal Chem) with a wide-range standard curve (0.1–12.8 ng/mL standard curve option) and read on the Epoch plate reader (Agilent).

### 5-HT quantification.

Plasma 5-HT level was quantified using ELISA (IM1749; Beckman Coulter) according to the manufacturer’s instructions. For tissue 5-HT quantification, pulverized tissues were transferred to 0.2 N perchloric acid and mechanically homogenized (Tissue Lyzer II; QIAGEN). After homogenization, samples were centrifuged at 10,000*g* to pellet insoluble debris and draw off supernatant. Transferred supernatant was neutralized with 1 M borate buffer. The 5-HT ELISA was performed with neutralized tissue extracts to quantify 5-HT (IM1749).

### Total gastrointestinal transit time assay.

A 6% carmine red solution was prepared by mixing 0.5% methylcellulose (Sigma) solution with carmine red powder (Sigma) and autoclaved. Mice were transferred to clean cages without bedding right after the dark cycle ended. To determine total gastrointestinal transit time, mice were gavaged with prepared carmine red solution and observed until they produced first red feces with carmine red.

### Fecal bomb calorimetry.

Mice were transferred to wire-bottom cages (Labcorp) and given 3 days for acclimation to the new environment. After acclimation, fecal samples were collected in 50 mL tubes for 5 days and stored at –20°C until analysis. During the collection, body mass, diet intake, and fecal weight were measured every day. Fecal samples were freeze-dried using a FreeZone Bulk Tray Dryer (Labconco) and ground using a coffee grinder. Fecal sample energy was measured via isoperibol oxygen bomb calorimetry using the Parr 6200 calorimeter with a Parr 6510 water handling system (Parr Instrument Co.).

### Histological analysis.

Dissected iWAT, eWAT, and liver tissue were fixed in 10% aqueous buffered zinc formalin (Z-fix; Anatech), embedded in paraffin, sectioned (5 μm), and stained with H&E. For CLS quantification, paraffin-embedded sections were deparaffinized with xylene and rehydrated with ethanol. Citrate buffer (antigen unmasking solution; Vector Laboratories) was used for antigen retrieval. Sections were blocked with the solution containing 5% BSA and 0.1% Tween 20 for 1 hour at room temperature. After blocking, slides were washed with PBS and incubated with primary antibody for Galectin-3 Polyclonal Antibody (1:1,000; Cedarlane CL8942AP) overnight on 4°C. After the overnight incubation, slides were washed with PBS 3 times and incubated with rabbit anti-mouse biotinylated IgG secondary antibody (1:500; Vector Laboratories; BA-9200-1.5) for 1 hour at room temperature. After washing with PBS 3 times, slides were incubated with VECTASTAIN Elite ABC HRP reagent (Vector Laboratories) for 30 minutes. Lastly, slides were developed with ImmPACT DAB EqV (Vector Laboratories), washed, and counterstained with hematoxylin. Digital images were acquired at ×10 or ×20 magnification (DX51 light microscope; Olympus). Average adipocyte size or GAL-3–positive area was quantified using ImageJ software (NIH).

### Hepatic TG quantification.

Liver tissues were bead-homogenized in RIPA buffer using the Tissue Lyzer II (QIAGEN), and Nonidet P-40 substitute (Sigma) was added to make 5% (w/v) homogenates. TGs were solubilized by heating homogenates to 95°C for 5 minutes and then cooling to room temperature, with this process repeated twice. Extracted hepatic TG was quantified using a commercially available kit (MAK266; Sigma) following the manufacturer’s instructions. TG content was normalized to the total protein concentration in homogenates and measured using a BCA Protein Assay Kit (Thermo Fisher Scientific).

### Mature adipocyte isolation and primary cell culture.

Dissected single depots of eWAT from C57BL/6J mice (The Jackson Laboratory) were minced with sharp razor blades and immediately put in DMEM with high glucose and HEPES (Gibco) with 2% BSA and 500 nM adenosine. eWAT homogenates were digested with collagenase type 1 (1 mg/mL) for 30 minutes on a shaking incubator (37C°). Digested adipocytes were filtered through a 250 μm cell strainer and washed with DMEM. After short 500*g* centrifugation, adipocytes were washed again with DMEM, then centrifuged at 500*g* for 15 minutes to separate mature adipocytes and SVFs. Separated adipocytes and SVFs were transferred to Qiazol (QIAGEN) and frozen at –80°C for subsequent RNA analysis. For primary adipocyte culture, the pelleted stromal vascular cells were resuspended in DMEM containing 10% FBS and seeded in 24-well plates for adipogenic differentiation. All experiments involving primary adipocytes were conducted after they were fully differentiated.

### Human sample analysis.

An abdominal subcutaneous adipose tissue biopsy was collected from each volunteer under overnight fasting conditions using a sterile technique and local anesthesia. Arterialized venous blood samples were collected from each volunteer under overnight fasting conditions for measurement of fasting plasma insulin concentrations, which were measured with a chemiluminescent immunoassay (Sanofi Diagnostics Pasteur). Subcutaneous adipose tissues (50 mg) with Qiazol were mechanically bead-homogenized (Tissue Lyzer II). Total RNA was extracted (RNeasy Mini columns; QIAGEN) from tissue homogenates, and RNA purity and concentration were quantified with a spectrophotometer (Nanodrop 100; Thermo Fisher Scientific). cDNA was generated with 1 μg of RNA by reverse transcription (High-Capacity cDNA Reverse Transcription Kit; Applied Biosystems). qPCR was performed using SYBR Green (PowerUp SYBR Green Master Mix; Applied Biosystems) on a QuantStudio 6 Flex real-time PCR system (Applied Biosystems). RT-PCR Ct values were normalized and analyzed with the 2–ΔΔ CT method. Forward and reverse primer sequences are listed in Supplemental Table 1.

### Immunoblotting analysis.

Liver tissue, muscle tissue, BAT, and eWAT (20~100 mg) were bead-homogenized with RIPA buffer (Thermo Fisher Scientific) plus phosphatase and protease inhibitor (Invitrogen). Total protein concentration of supernatants was measured with a BCA protein assay (Thermo Fisher Scientific). Cell lysate with equal amount of protein was mixed with 4× NuPAGE LDS sample buffer (Thermo Fisher Scientific) with 50 mM DTT and heated at 85°C for 5 minutes. Protein extracts were separated by 4%–20% SDS-PAGE gel (Bio-Rad), transferred to a nitrocellulose membrane (Bio-Rad), and blocked with 5% nonfat milk with TBS with 0.1% Tween 20 (TBST). The membranes were incubated with the following primary antibodies: anti–phospho-Akt-Ser473 (Cell Signaling 9271; 1:1,000), anti-Akt (Cell Signaling 9272; 1:1,000), anti-TPH2 (Abcam; EPR19191; 1:1,000), anti–β-actin (Proteintech 60008-1-Ig; 1:5,000), and anti-UCP1 antibody (Cell Signaling 14670; 1:1,000). After washing with TBST 3 times, membranes were incubated with IRDye 800CW Dye-Labeled or IRDye 680RD Dye-Labeled Secondary Antibody (LICOR). Fluorescence signal was detected and analyzed using the iBright imaging system (Thermo Fisher Scientific).

### Insulin signaling in vivo.

Mice were fasted overnight and intraperitoneally injected with insulin (10 IU/kg body weight; Humulin R U-100, Eli Lily; diluted with sterile PBS). After 10 minutes, liver tissue, eWAT, and mixed gastrocnemius muscle were collected and immediately frozen with liquid nitrogen. Collected specimens were stored at –80°C for further Western blot analysis.

### RNA isolation and qPCR.

iWAT, eWAT, BAT, and liver tissue (20~100 mg) were submerged in Qiazol and mechanically bead-homogenized (Tissue Lyzer II). From tissue homogenates, total RNA was extracted (RNeasy Mini columns), and RNA purity and concentration were quantified with a spectrophotometer (NanoDrop 100; Thermo Fisher Scientific). cDNA was generated with 1 μg of RNA by reverse transcription (High-Capacity cDNA Reverse Transcription Kit). qPCR was performed using SYBR Green (PowerUp SYBR Green Master Mix) on a QuantStudio 6 Flex real-time PCR system. RT-PCR Ct values were normalized and analyzed with the 2–ΔΔ CT method. Forward and reverse primer sequences are listed in Supplemental Table 1.

### Statistics.

All statistical analyses were conducted using GraphPad Prism 9 software. FDR-adjusted 2-tailed Student’s *t* test, 1-way ANOVA with Tukey’s multiple-comparison test, 2-way ANOVA with Dunnett’s test, or Welch and Brown-Forsythe ANOVA were performed to determine statistical differences between groups, as appropriate. Pearson’s correlation analysis was used to assess the relationships between fasting insulin levels and gene expression in humans. A *P* value of less than 0.05 was considered to be significant. All data were expressed as mean ± SEM.

### Study approval.

All of the experimental procedures were in accordance with standards and guidelines approved by the IACUC of Tufts University. The human adipose and blood samples were collected as part of Institutional Review Board–approved studies conducted at the Mayo Clinic in its Clinical Research Trials Unit. All volunteers were healthy and provided informed, written consent.

### Data availability.

Values for all data points in graphs are reported in the Supporting Data Values file.

## Author Contributions

BIP, ARR, MDJ, and ASG conceived the study and designed the methodology. BIP, ARR, RAW, YZ, KKB, SCF, and ASG performed the investigation. BIP and ASG wrote the original draft. BIP, KKB, MDJ, and ASG reviewed and edited the article. BIP and ASG acquired funding. ASG contributed resources. KAL performed the investigation and reviewed and edited the article. MDJ and ASG supervised the study.

## Supplementary Material

Supplemental data

Unedited blot and gel images

Supplemental table 1

Supporting data values

## Version 1 06/02/2025

In-Press Preview

## Version 2 07/15/2025

Electronic publication
