# Supplementary material for: Hyperinsulinemia-induced upregulation of adipocyte TPH2 contributes to peripheral serotonin production, metabolic dysfunction, and obesity
Source: J Clin Invest. 2025 May 30;135(14):e190765. doi: 10.1172/JCI190765 (PMC12259259; doi:10.1172/JCI190765)
Supplement: Supplemental data [file jci-135-190765-s167.pdf]

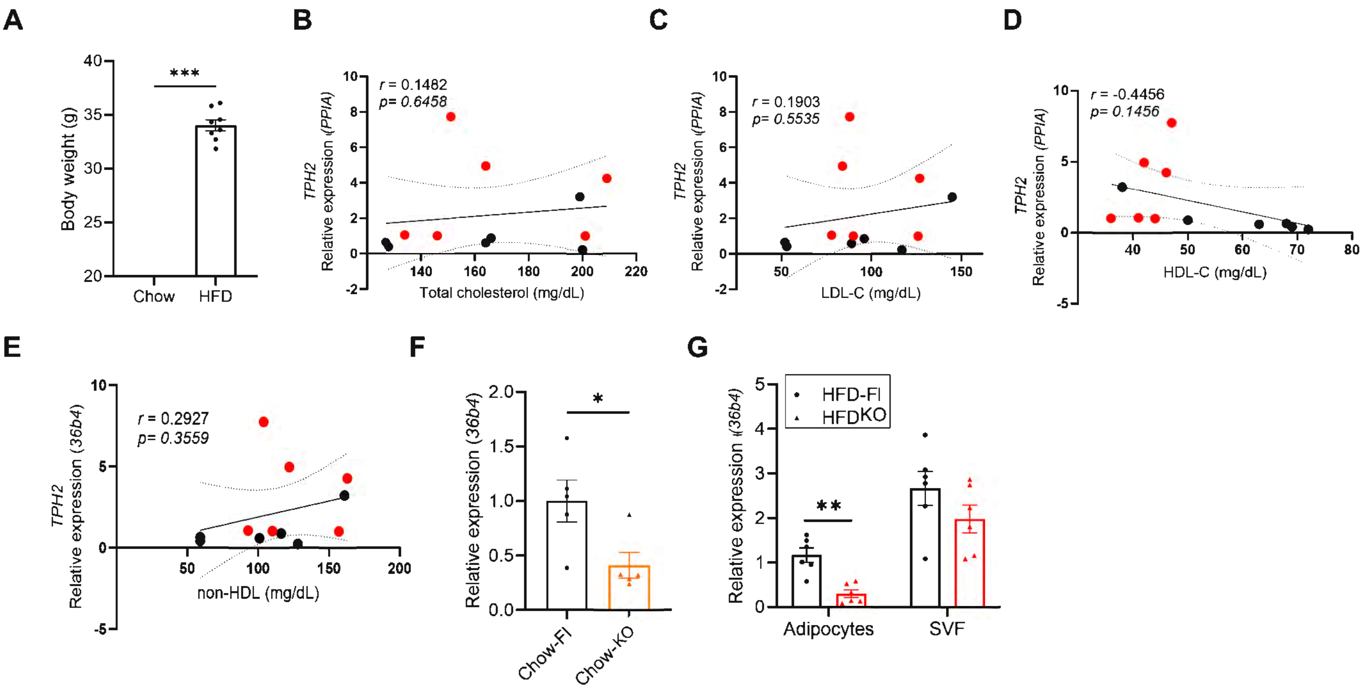

Supplemental Fig. 1 Relationship between *TPH2* expression of human subcutaneous WAT and cholesterol levels, and genetic deletion of adipocyte TPH2 in mice, related to Fig. 1 and Fig. 2.

(A) Body weights of C57BL/6J mice after 6 weeks of chow or HFD feeding. (n=4 for Chow, n=8 for HFD)

(B-E) Correlation between TPH2 expression in human subcutaneous fat and total cholesterol (B), LDL (C), HDL (D) and non-HDL (E) levels of lean and obese individuals (n=6 per group), Pearson's r correlation coefficient with corresponding p-values.

(F) Relative expression levels of *Tph2* in eWAT from chow-fed mice. (n=8 per group)

(G) Tph2 mRNA levels of isolated mature adipocytes and SVF in HFD-fed mice. (n=6 per group)

Data are presented as mean ± SEM. For statistical analysis, two-tailed Student's t test were used, *p < 0.05, **p < 0.01, ***p < 0.001.

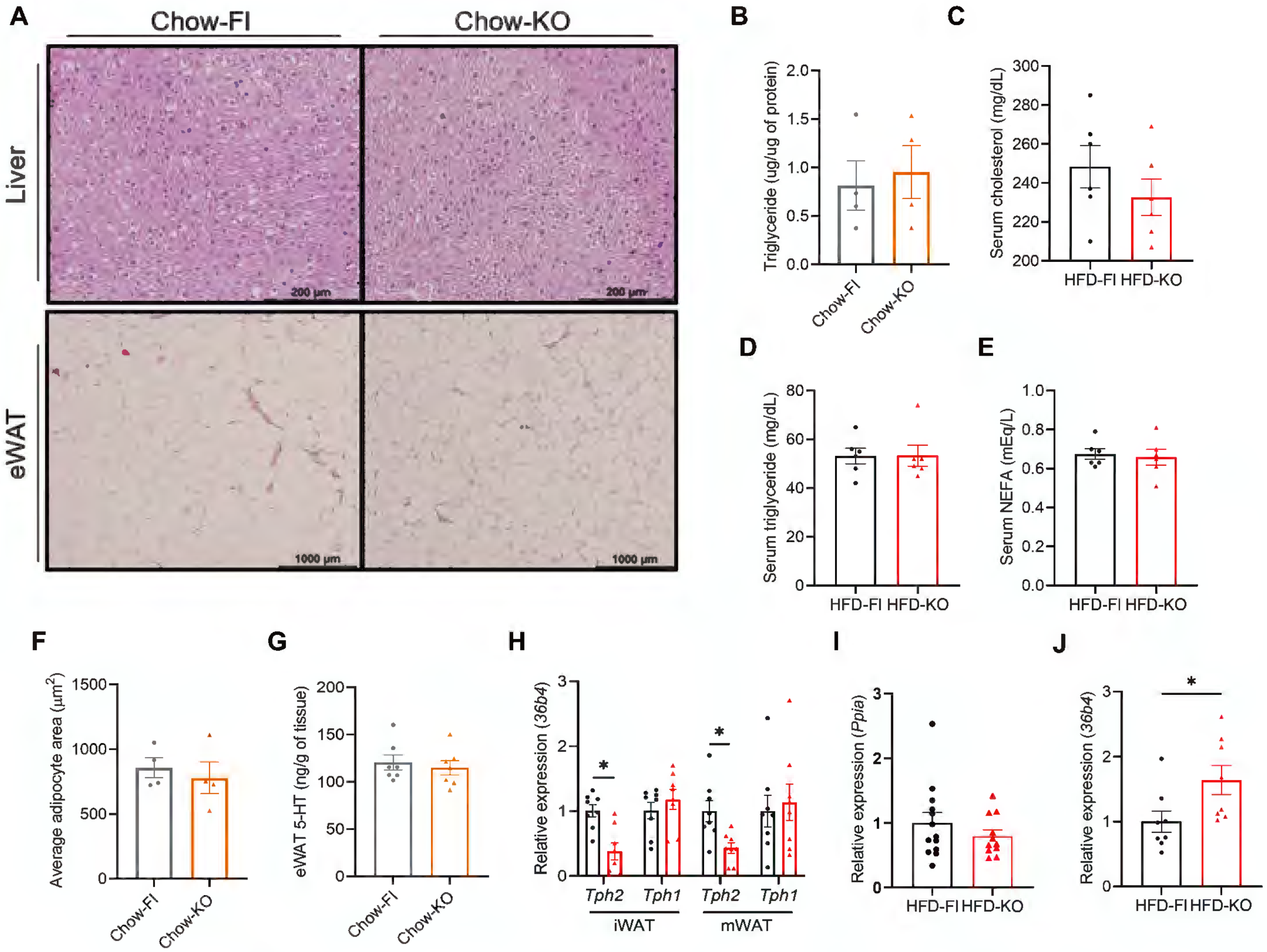

Supplemental Fig. 2 The loss of adipocyte TPH2 did not alter liver and eWAT physiology in Chow-fed mice, related to Fig. 3.

(A) Representative images of H&E stained liver and eWAT after 12 weeks of chow feeding. (n=5 per group)
(B) TG levels in liver of mice after 12 weeks of chow feeding. (n=5 per group)
(C-E) Serum levels of cholesterol (C), TG (D) and NEFA (E) after 12 weeks of HFD feeding. (n=6 per group)
(F) Average adipocyte size of eWAT after 12 weeks of chow feeding. (n=5)
(G) 5-HT levels of eWAT from mice fed chow for 12 weeks. (n=7 per group)
(H) mRNA levels of *Tph2* and *Tph1* in iWAT and mWAT after 12 weeks of HFD feeding . (n=8 per group)
(I) mRNA levels of *Tph1* in ileum 12 weeks of HFD feeding . (n=11~13 per group)
(J) mRNA levels of *Adipoq* of eWAT after 12 weeks of HFD feeding. (n=8 per group)
Data are presented as mean ± standard error of mean (SEM). For statistical analysis, two-tailed Student's t test (A-F) was used, *p < 0.05, **p < 0.01, ***p < 0.001

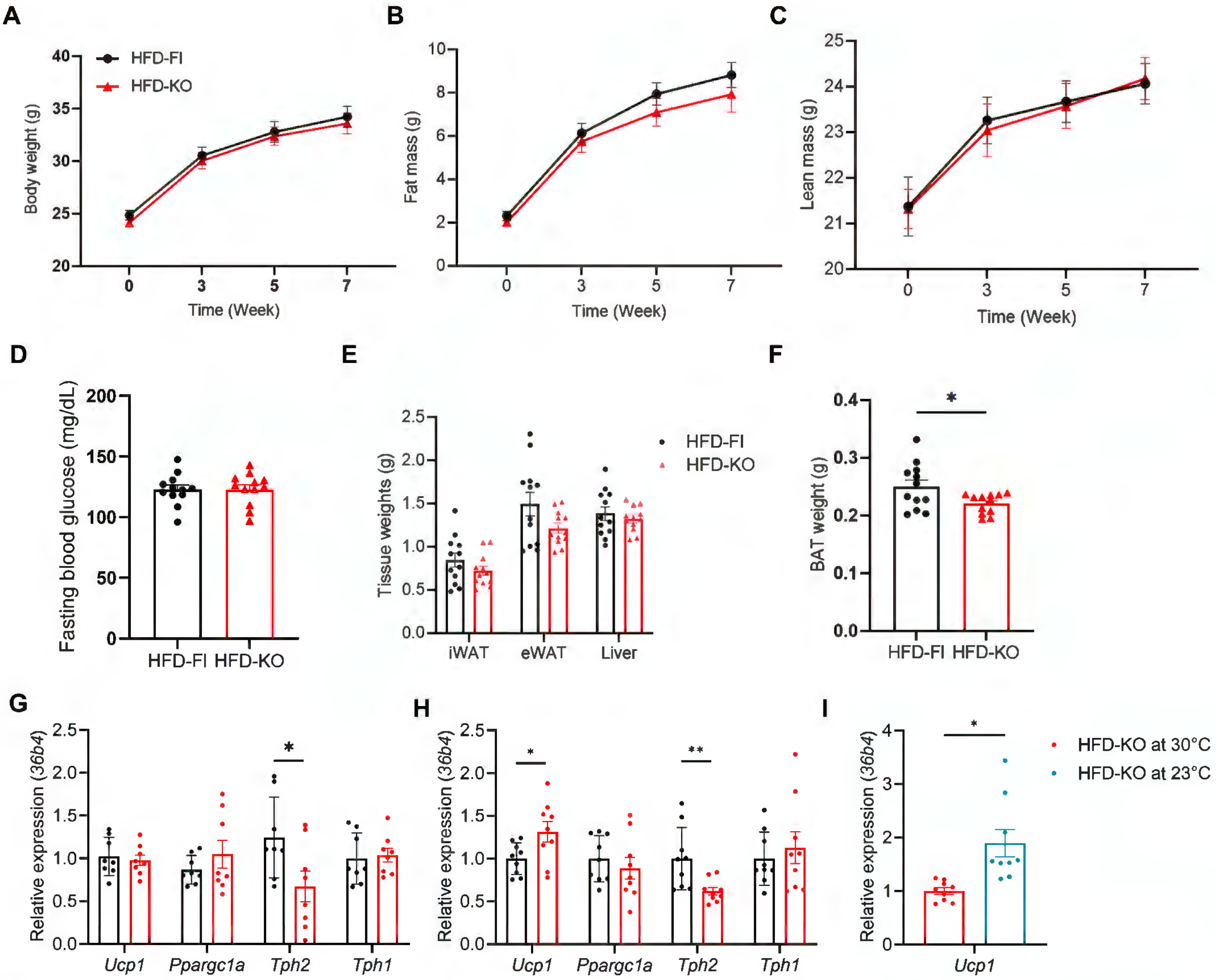

Supplemental Fig. 3 The effect of thermoneutrality on HFD-fed mice with adipocyte-specific TPH2 ablation, related to Fig. 4.

(A-C) Time course body weight (C), fat (D) and lean mass (E) following HFD feeding and thermoneutral housing (n=12 per group).
(D) Fasting blood glucose. (n=12 per group)
(E, F) Tissue weights iWAT, eWAT and liver (E) and BAT(F). (n=12 per group)
(G) mRNA levels of *Ucp1, Ppargc1a*, *Tph2* and *Tph1* in iWAT after 8 weeks of HFD feeding and thermoneutral housing. (n=9 per group)
(H) mRNA levels of *Ucp1, Ppargc1a*, *Tph2* and *Tph1* in BAT after 8 weeks of HFD feeding and thermoneutral housing. (n=9 per group)
(I) mRNA levels of *Ucp1* in BAT from HFD-KO mice housed in either room temperature or thermoneutrality for 8 weeks. (n=9 per group)
Data are presented as mean ± standard error of mean (SEM). For statistical analysis, two-tailed Student's t test (A-I) was used, *$p < 0.05$, **$p < 0.01$

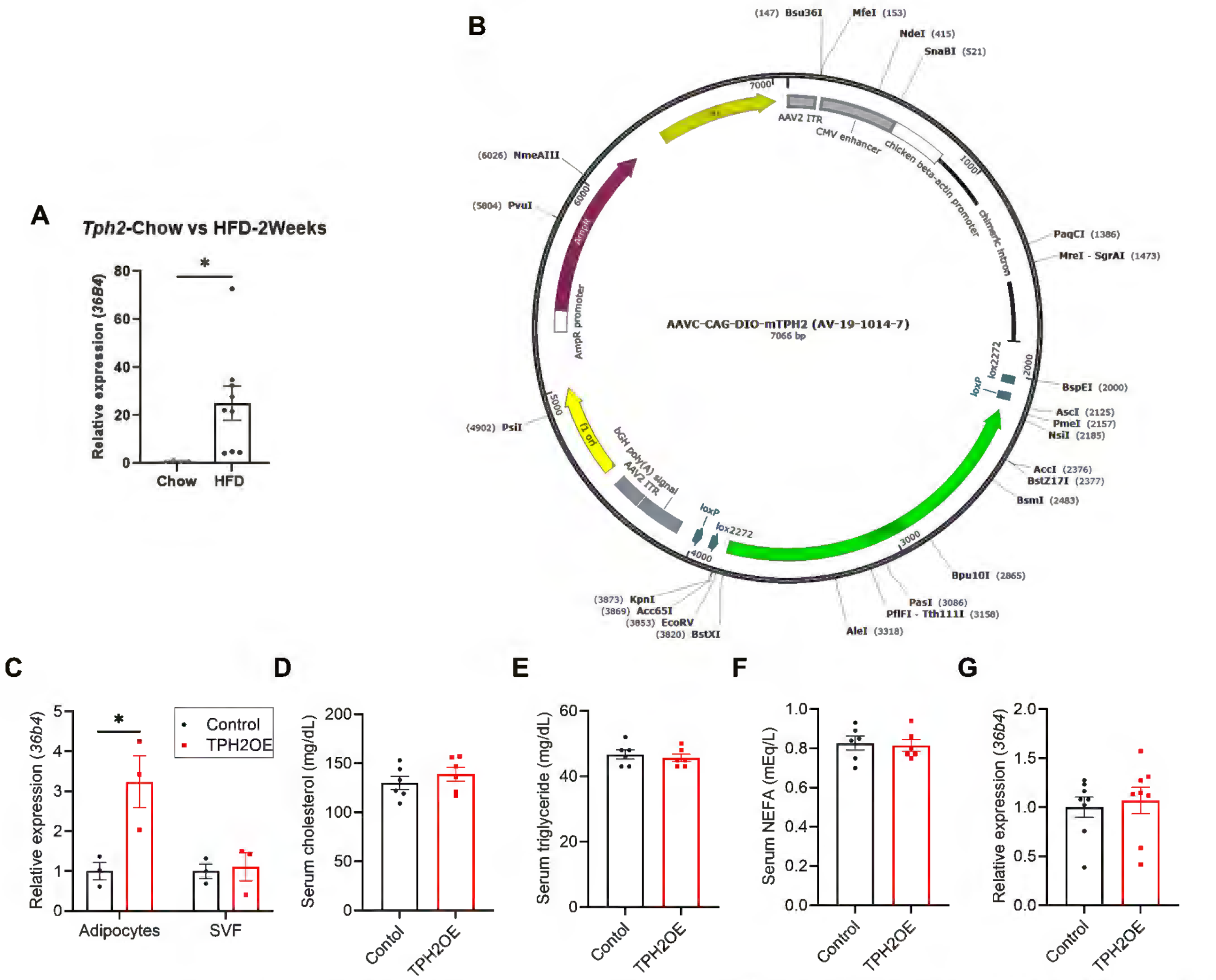

Supplemental Fig. 4 Overexpression of adipocyte TPH2 did not alter serum lipid profile. Related to Fig. 5,6.

(A) Relative expression of *Tph2* in eWAT of C57BL6/J mice either on chow or HFD for 2 weeks. (n=4 for chow and n=8 for HFD)
(B) Circular map for AAV-TPH2.
(C) mRNA levels of *Tph2* in isolated adipocytes and SVF from eWAT, 20 weeks after AAV-TPH2 injection. (n=8 per group)
(C-F) Serum levels of cholesterol (D), TG (E) and NEFA (F), 20 weeks after AAV-TPH2 injection. (n=6 per group)
(G) mRNA levels of *Adipoq* of eWAT, 20 weeks after AAV-TPH2. (n=8 per group)
Data are presented as mean ± standard error of mean (SEM). For statistical analysis, two-tailed Student's t test (A-G) was used, *p < 0.05, **p < 0.01, ***p < 0.001

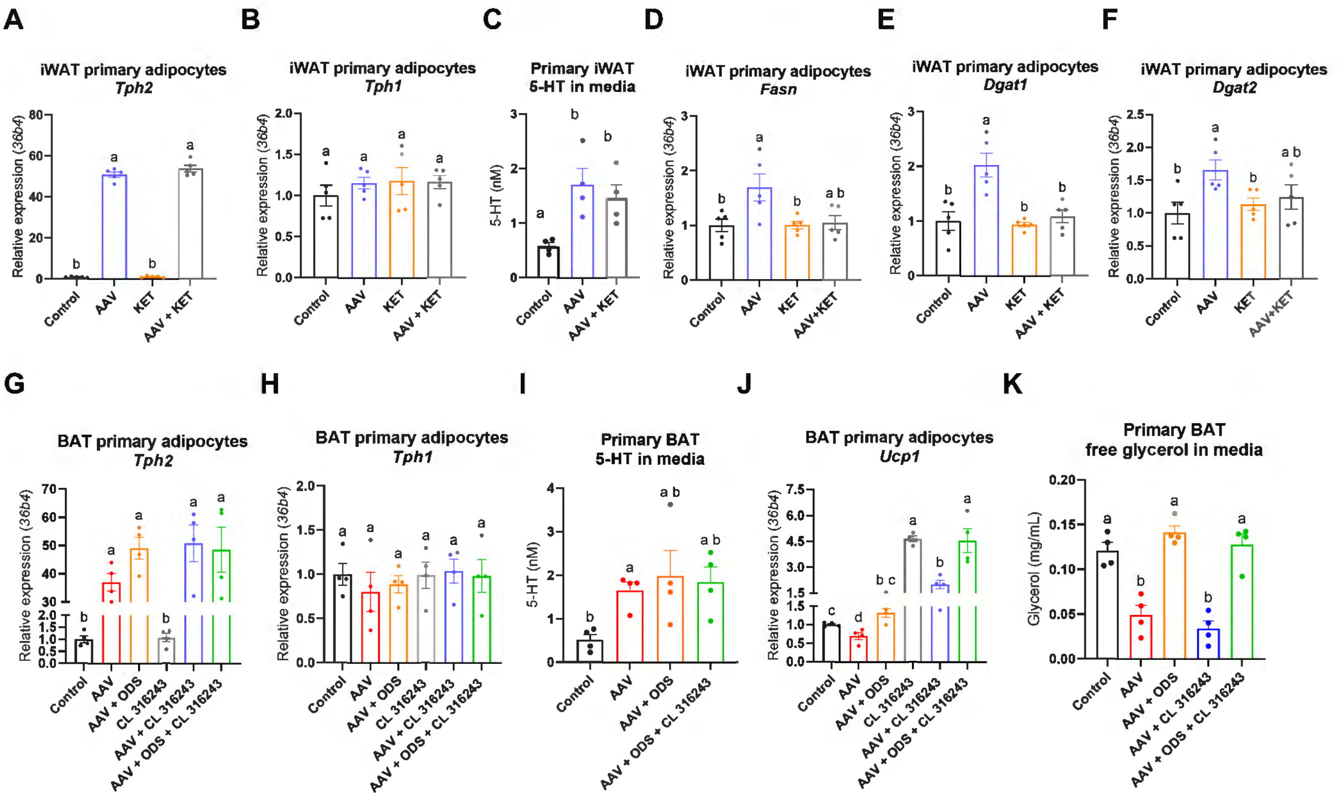

Supplemental Fig. 5 The effect of TPH2 overexpression in differentiated primary white and brown adipocytes using TPH2-AAV. Related to Fig. 7, 8.

(A) mRNA levels of *Tph2* in differentiated white adipocytes with TPH2 overexpression and Ketanserin treatment (200 nM, n=5 per group).

(B) mRNA levels of *Tph1* in differentiated white adipocytes with or without TPH2 overexpression and Ketanserin treatment (200 nM, n=5 per group).

(C) 5-HT levels in culture media with or without TPH2 overexpression and Ketanserin treatment (200 nM, n=4 per group).

(D-F) mRNA levels of lipogeneic genes in differentiated white adipocytes with or without TPH2 overexpression and Ketanserin treatment (200 nM, n=5 per group).

(G) mRNA levels of *Tph2* in differentiated brown adipocytes with or without TPH2 overexpression, Ondansetron (1 μM) or CL 316243 (100nM) treatment, n=4 per group).

(H) mRNA levels of *Tph1* in differentiated brown adipocytes with or without TPH2 overexpression, Ondansetron (1 μM) or CL 316243 (100nM) treatment, n=4 per group).

(I) 5-HT levels in culture media with or without TPH2 overexpression, Ondansetron (1 μM) or CL 316243 (100nM) treatment, n=4 per group).

(J) mRNA levels of *Ucp1* in differentiated brown adipocytes with or without TPH2 overexpression, Ondansetron (1 μM) or CL 316243 (100nM) treatment, n=4 per group).

(K) Free glycerol levels in culture media with or without TPH2 overexpression, Ondansetron (1 μM) or CL 316243 (100nM) treatment, n=4 per group).

Data are presented as mean ± SEM. For statistical analysis, Welch and Brown-Forsythe ANOVA (A, B, D-K) or one-way ANOVA with Tukey's multiple comparison test (C), $^{*}p < 0.05$, $^{**}p < 0.01$, $^{***}p < 0.001$ were used, compact letter display indicates statistical difference between treatments, $p < 0.05$.

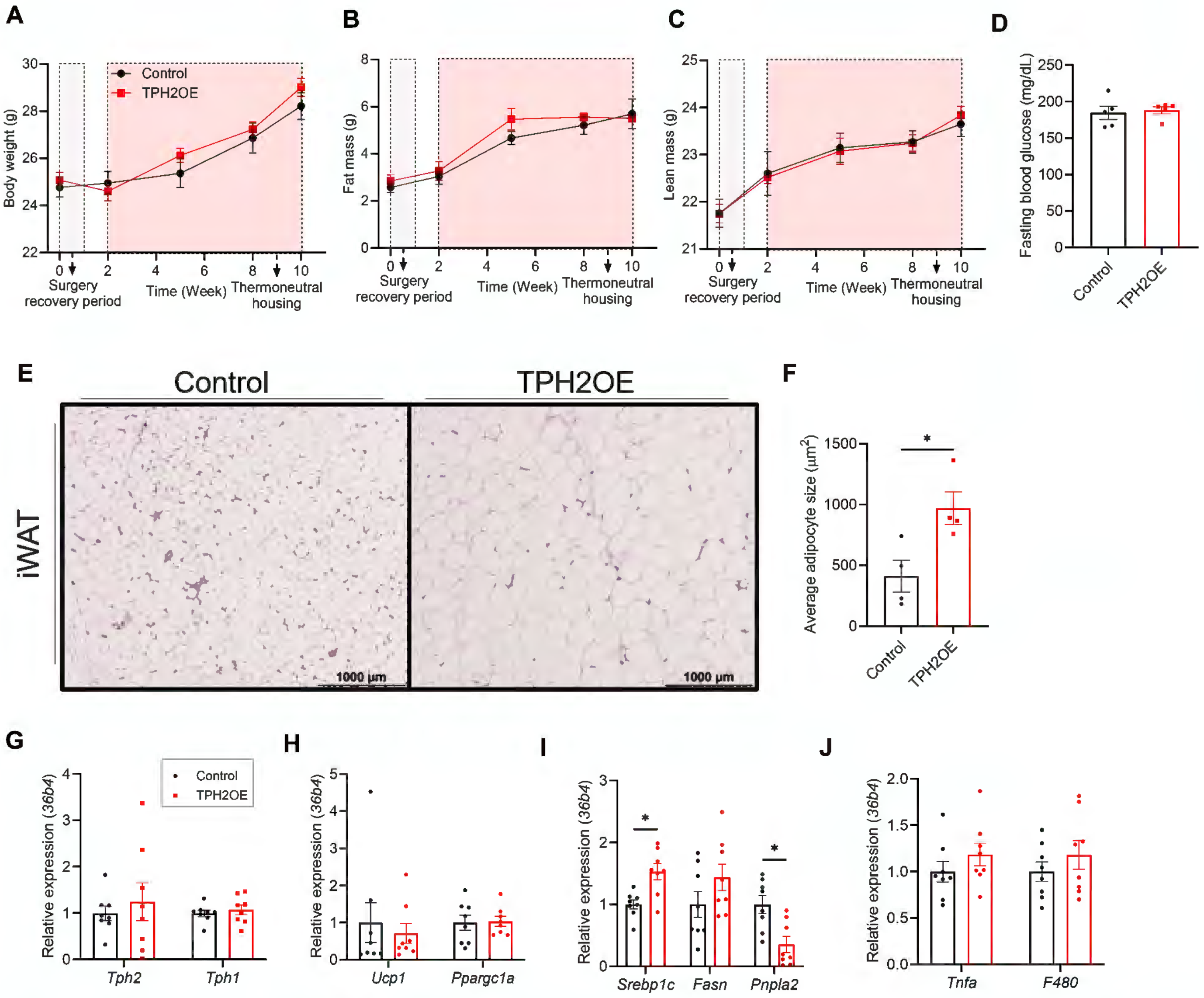

Supplemental Fig. 6 The effect of thermoneutrality on TPH2OE mice physiology and iWAT molecular profile. Related to Fig. 7.

(A-C) Time course body weight (C), fat (D) and lean mass (E) after AAV-TPH2 injection and thermoneutral housing (n=5 per group).
(E) Representative images of H&E stained iWAT, 20 weeks after AAV-TPH2 infection. (n=5 per group)
(F) Average adipocyte size of iWAT, 20 weeks after AAV-TPH2 injection. (n=5 per group)
(G) mRNA levels of *Tph2* and *Tph1* in iWAT, 20 weeks after AAV-TPH2 injection. (n=8 per group)
(H) iWAT mRNA levels of genes involved thermogenesis, 20 weeks after AAV-TPH2. (n=8 per group)
(I) iWAT mRNA levels of genes involved in lipid metabolism, 20 weeks after AAV-TPH2. (n=8 per group)
(J) iWAT mRNA levels of genes involved in proinflammatory pathway, 20 weeks after AAV-TPH2. (n=8 per group)
Data are presented as mean ± standard error of mean (SEM). For statistical analysis, two-tailed Student's t test was used, *p < 0.05, **p < 0.01, ***p < 0.001

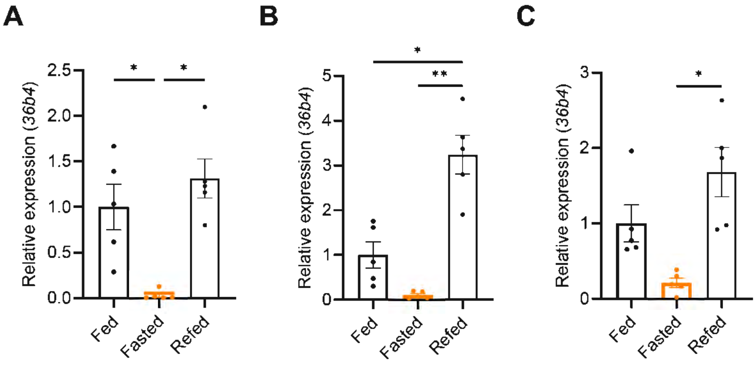

Supplemental Fig. 7 *Tph2* expression in adipose tissues from different prandial states, related to Fig. 8.

(A) Relative expression levels of *Tph2* in iWAT from chow-fed mice in different prandial states. (n=5 per group)
(B) Relative expression levels of *Tph2* in eWAT from chow-fed mice in different prandial states. (n=5 per group)
(C) Relative expression levels of *Tph2* in BAT from chow-fed mice in different prandial states. (n=5 per group)
Data are presented as mean ± SEM. For statistical analysis, two-tailed Student's t test were used, *p < 0.05, **p < 0.01, ***p < 0.001.
