# Supplementary material for: Hyperinsulinemia-induced upregulation of adipocyte TPH2 contributes to peripheral serotonin production, metabolic dysfunction, and obesity
Source: J Clin Invest. 2025 May 30;135(14):e190765. doi: 10.1172/JCI190765 (PMC12259259; doi:10.1172/JCI190765)

Full unedited blot for Figure 1E

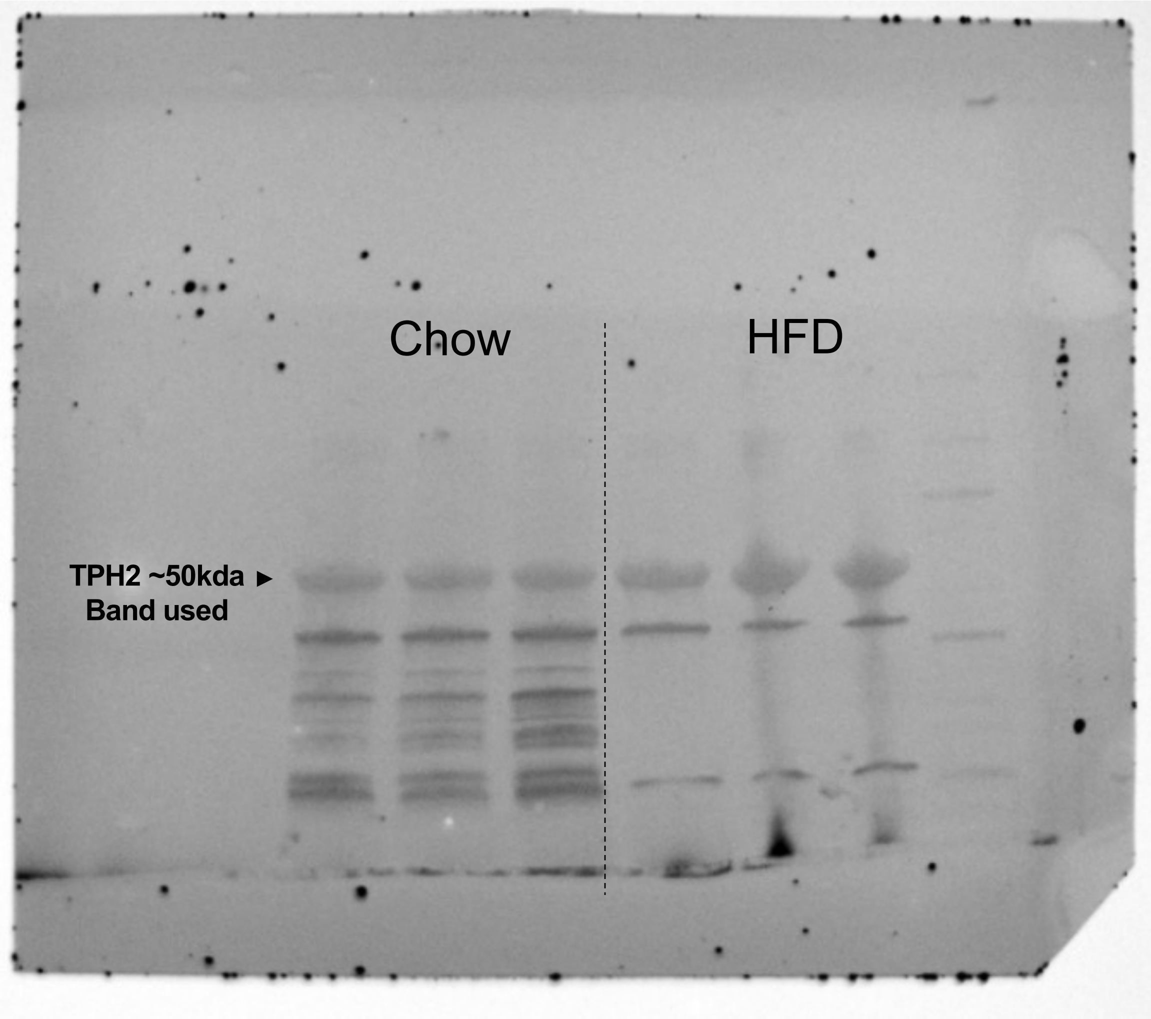

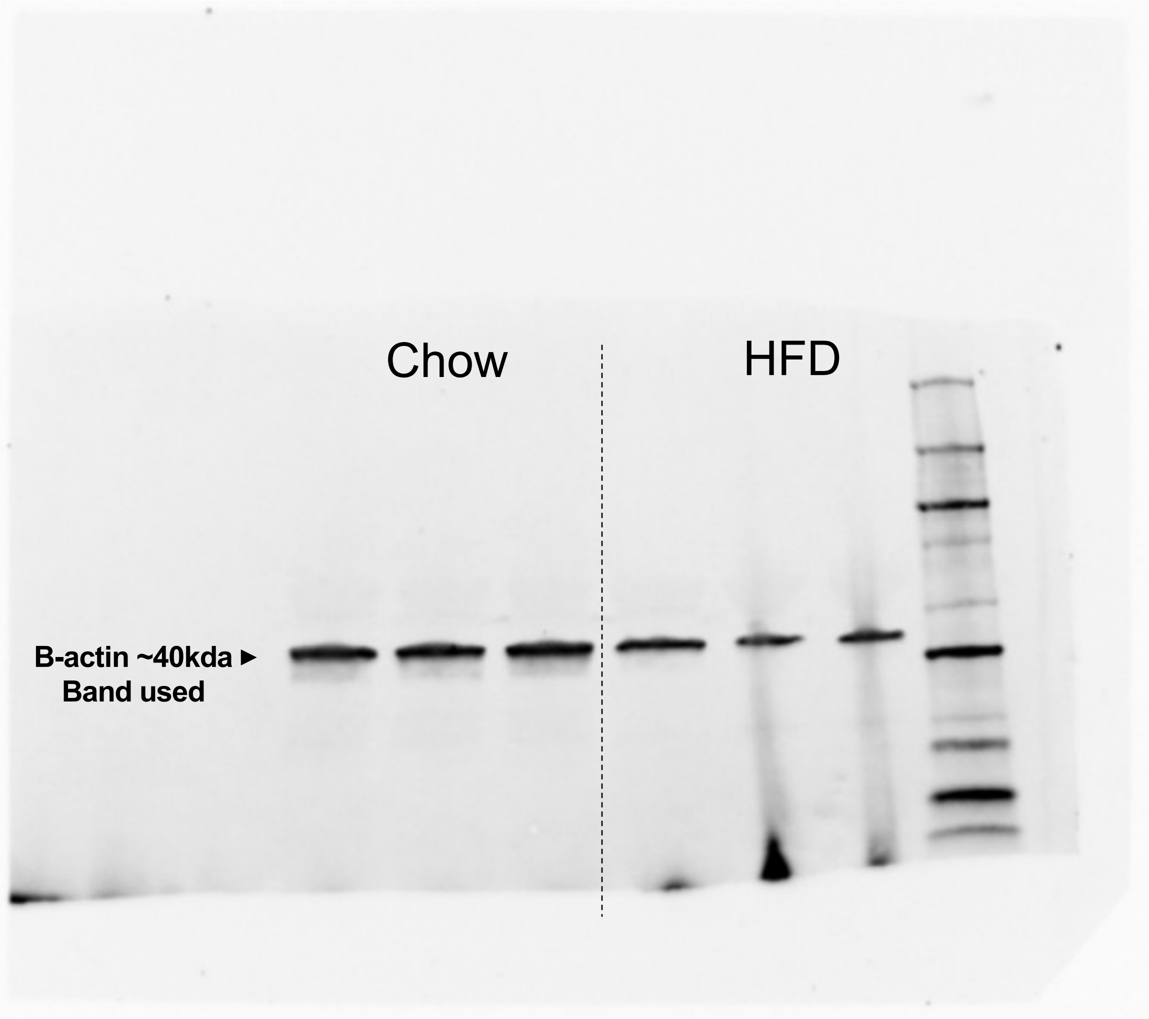

Full unedited blot for Figure 2N-Liver

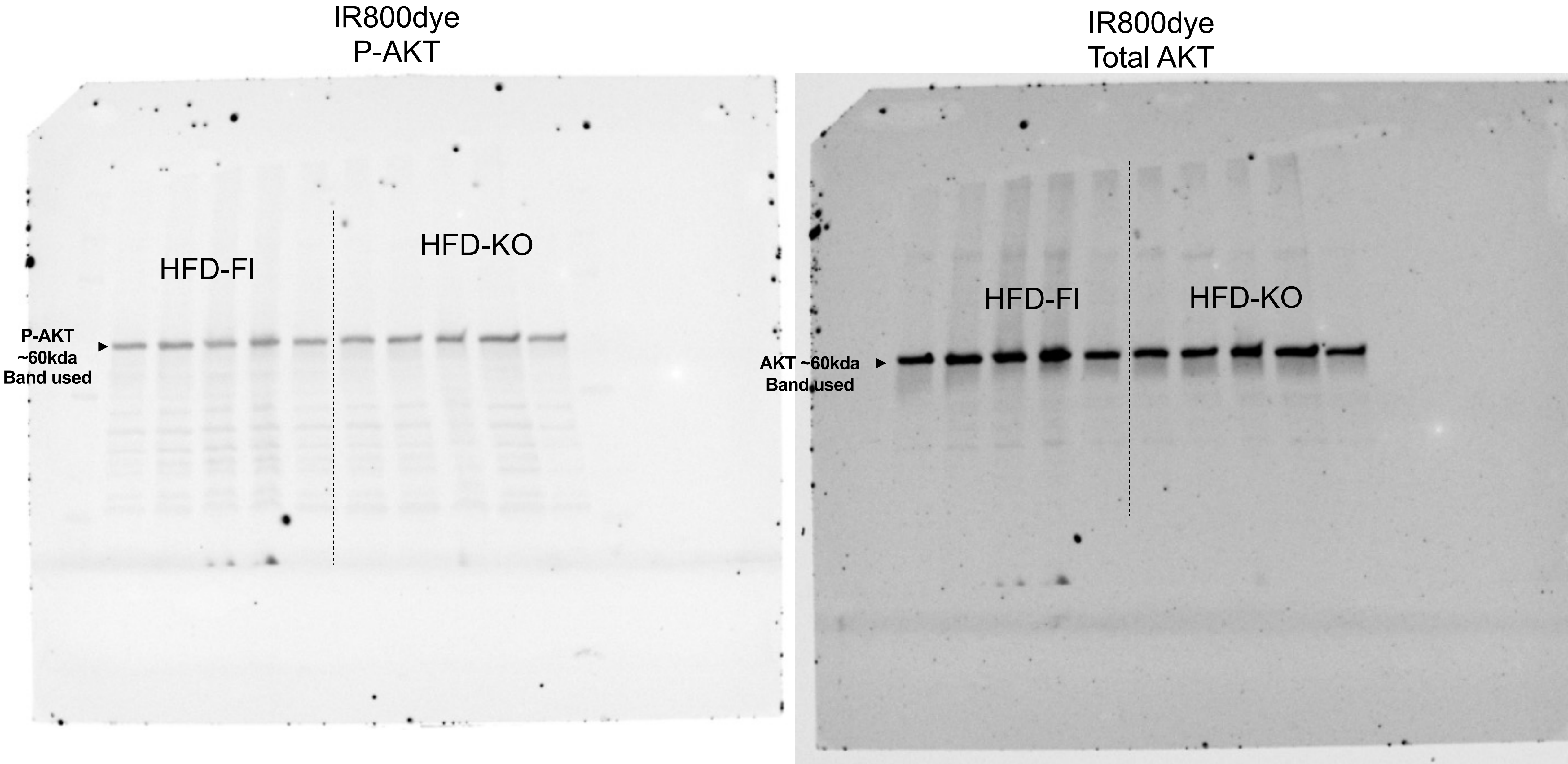

Full unedited blot for Figure 2N-eWAT

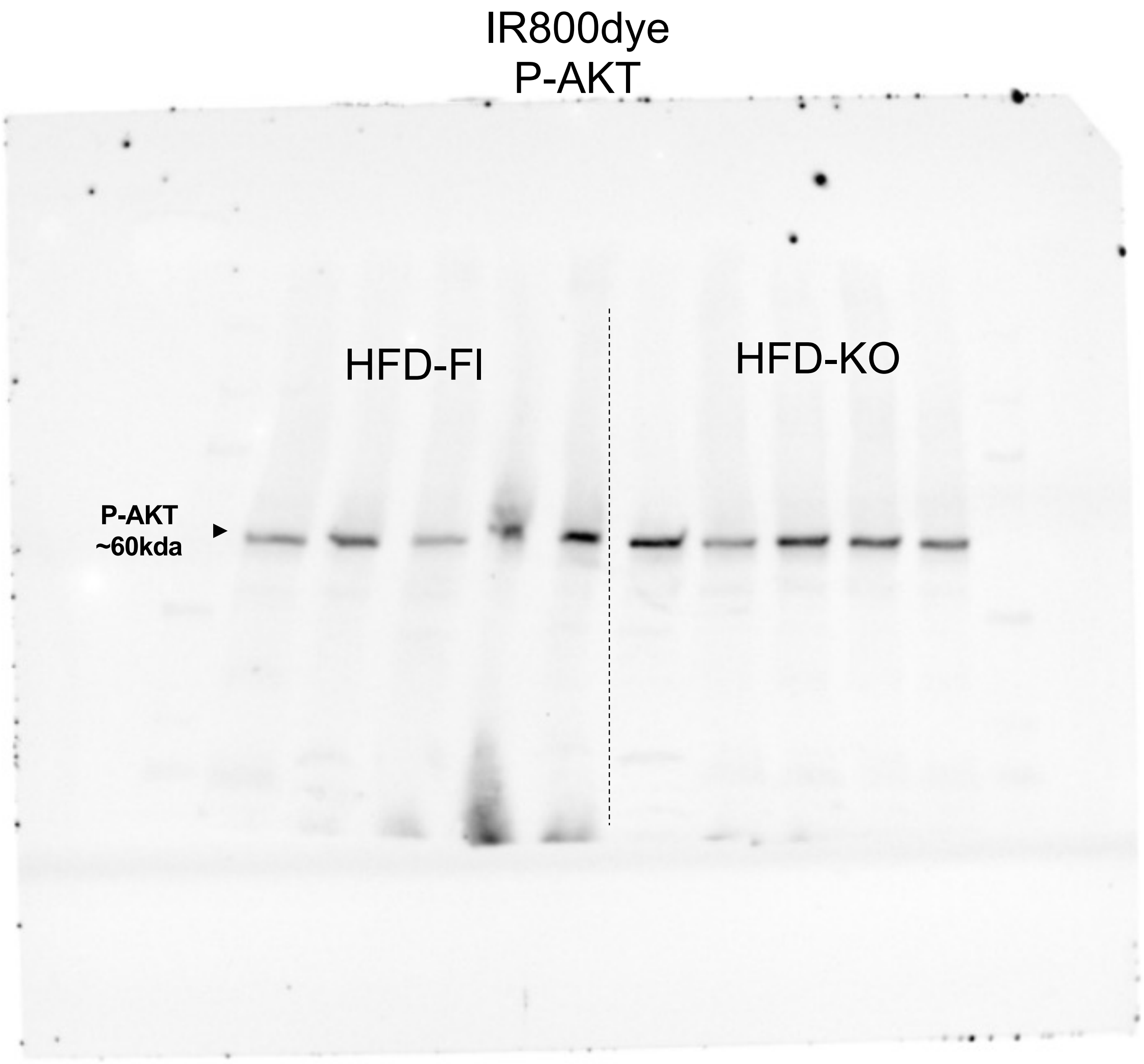

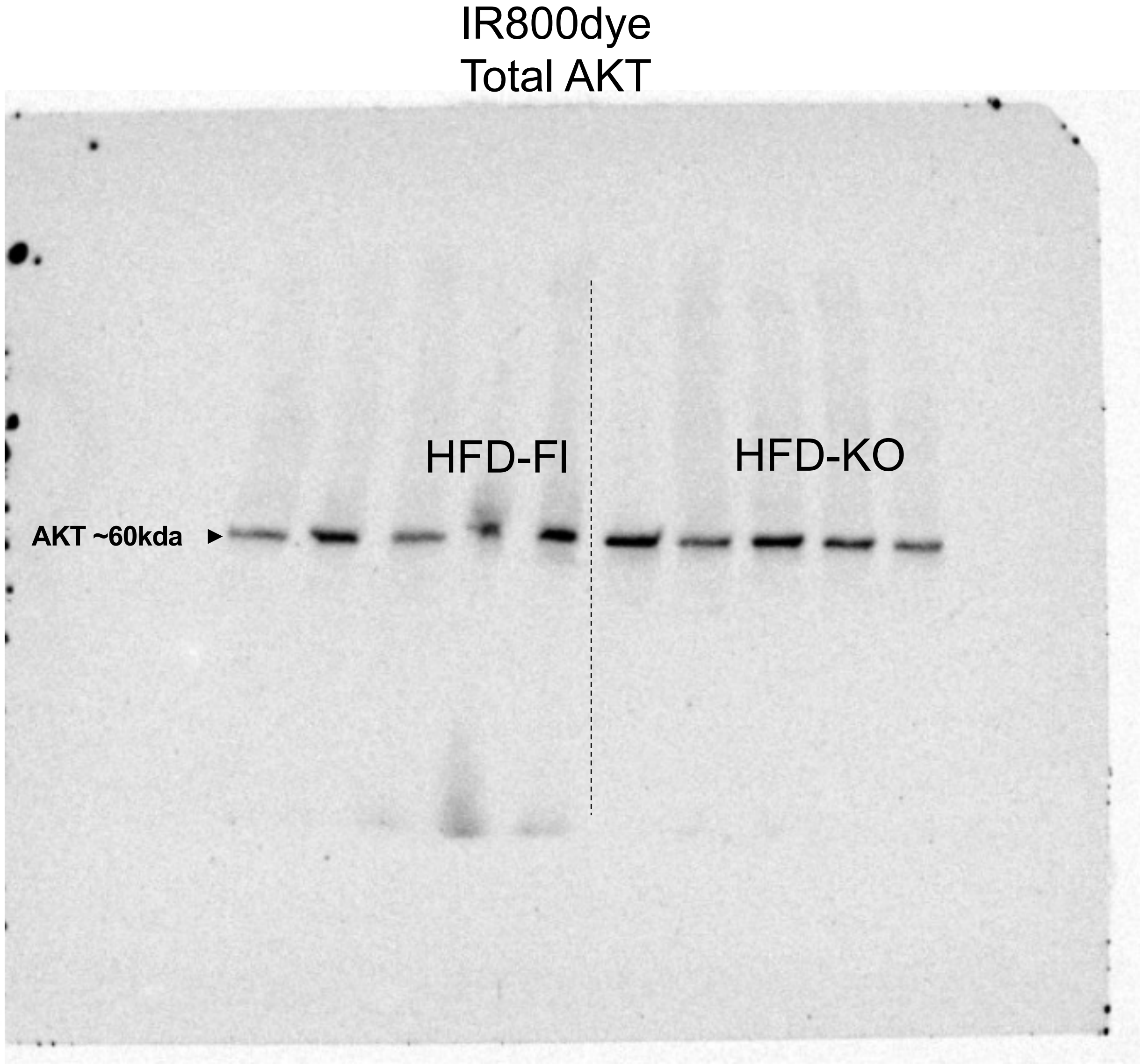

Full unedited blot for Figure 2N-Muscle

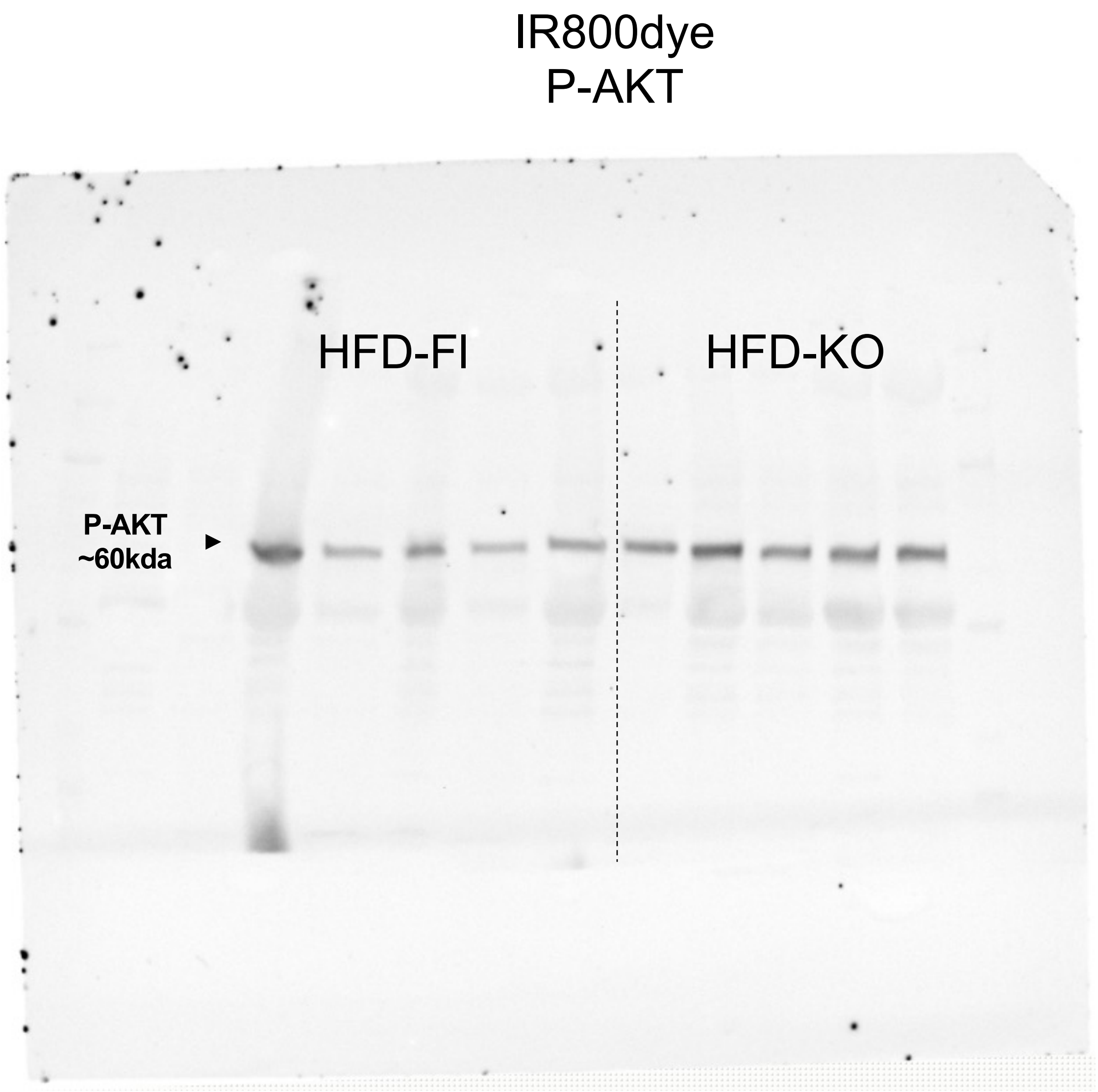

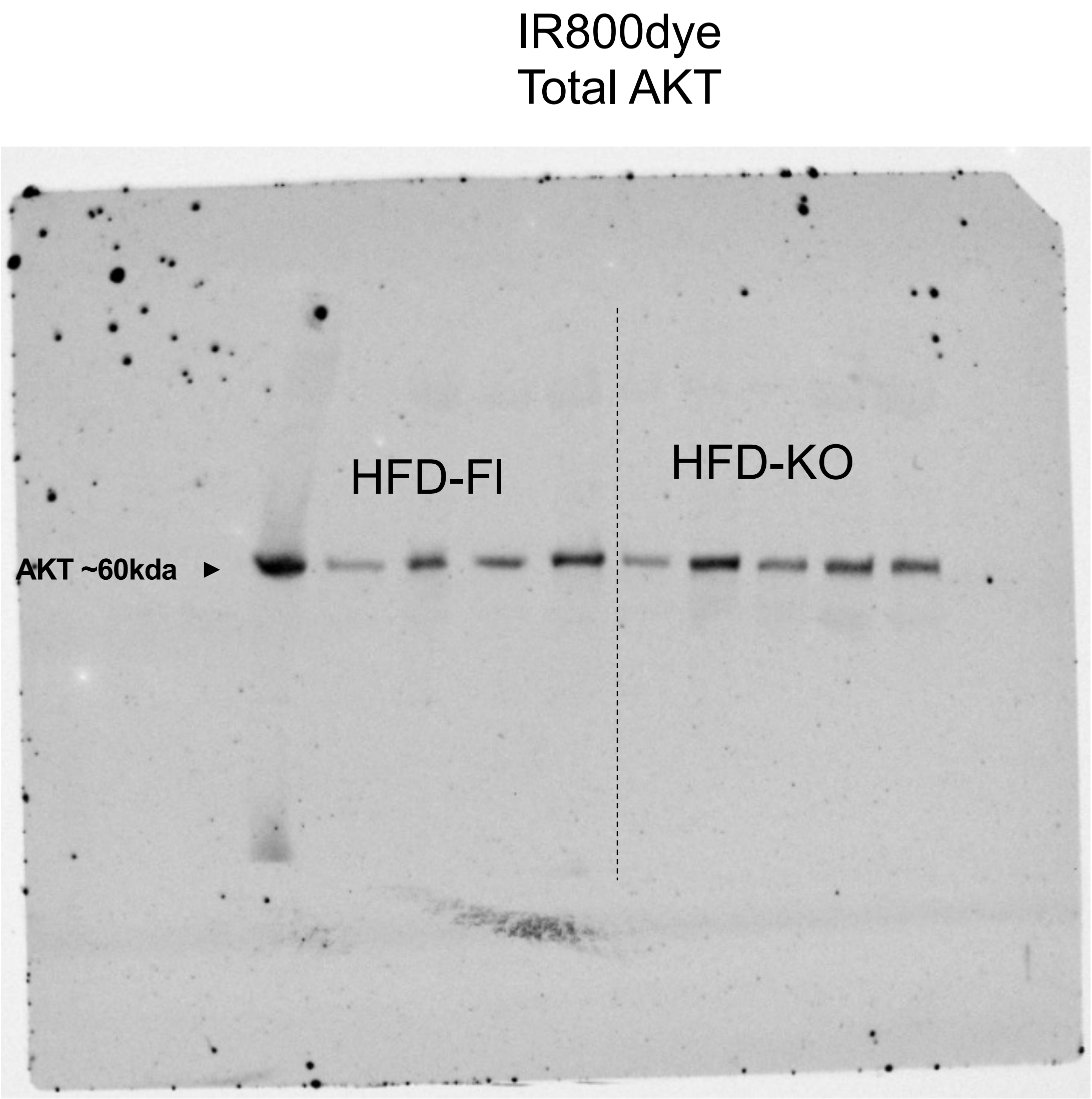

Full unedited blot for Figure 3I and 8A

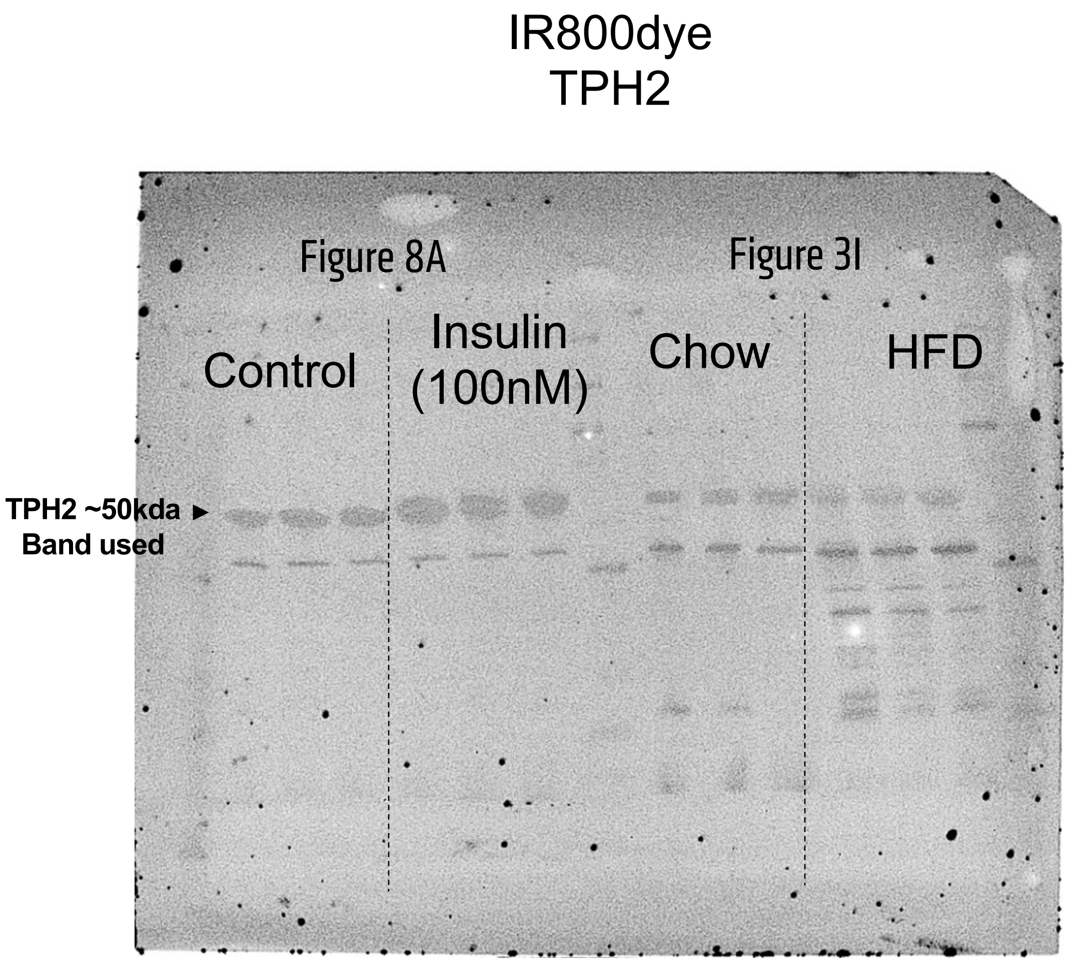

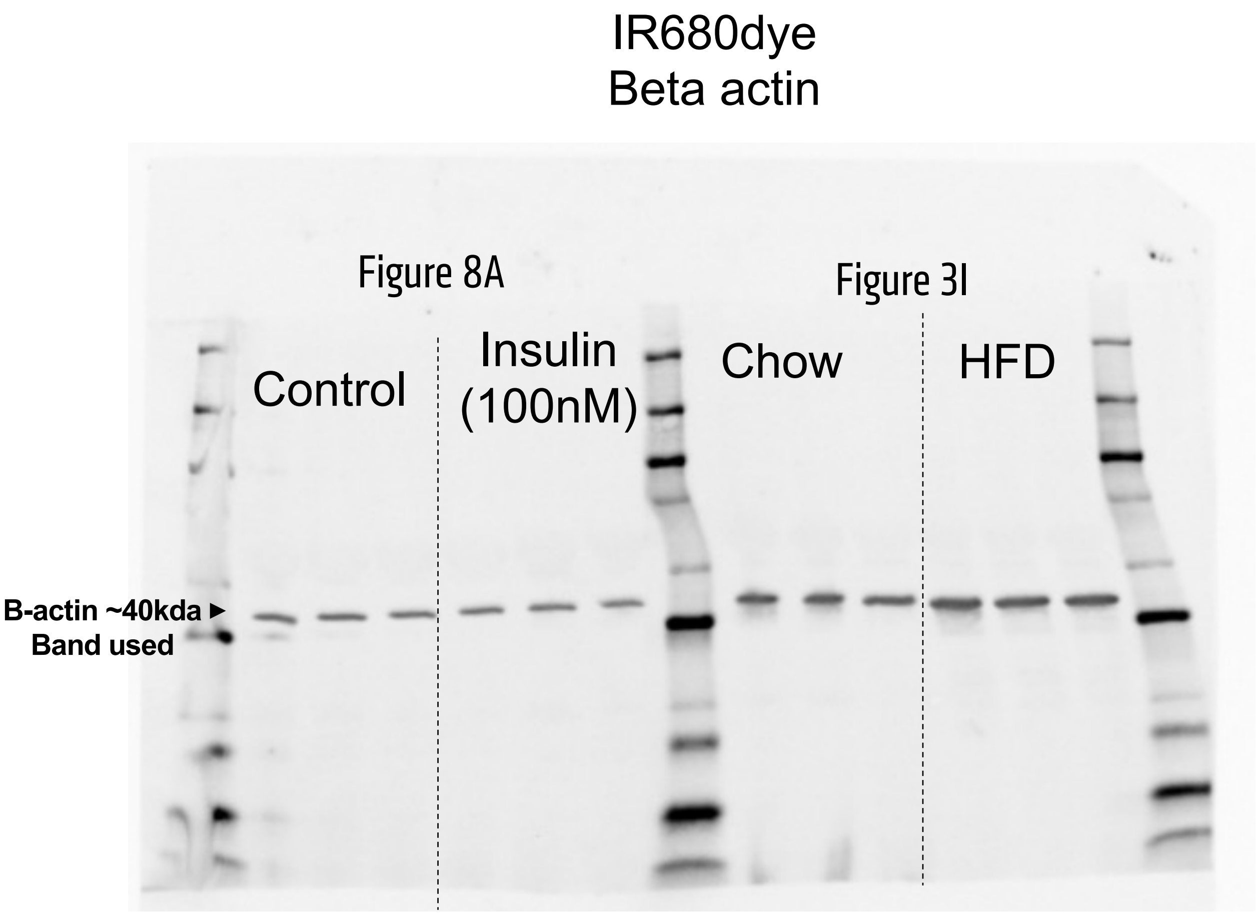

Full unedited blot for Figure 4G

IR800dye
UCP1

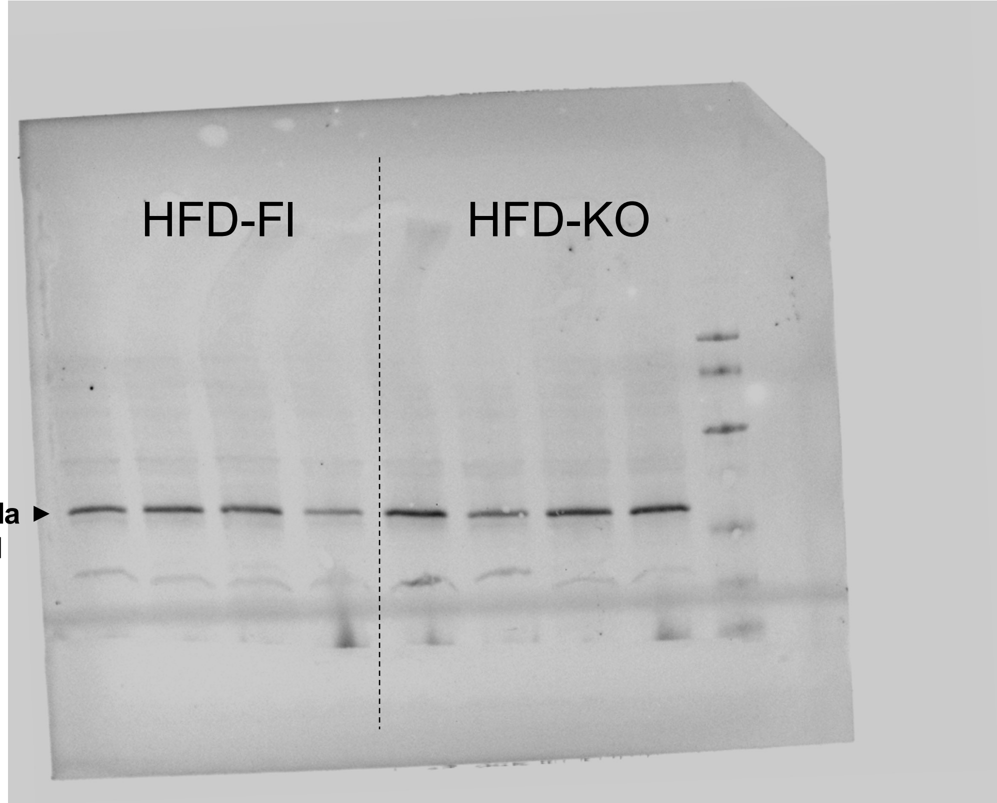

UCP1 ~30kda
Band used

IR680dye
Beta actin

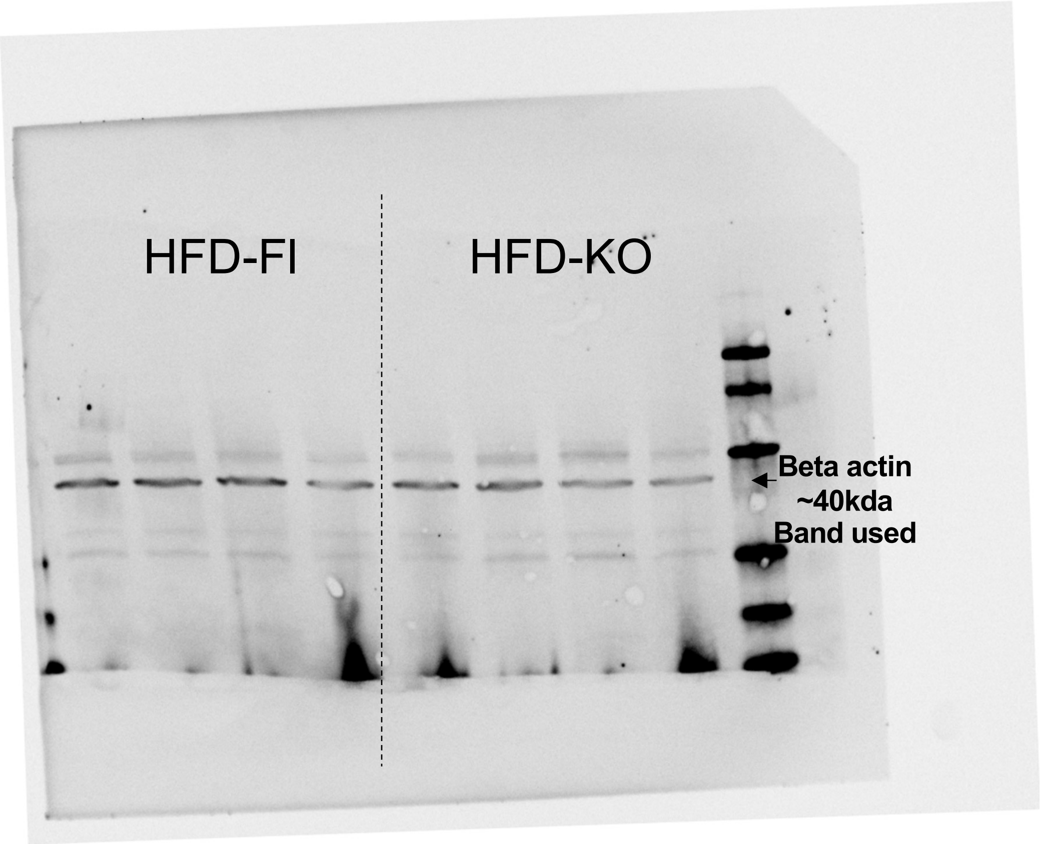

Full unedited blot for Figure 5R-Liver

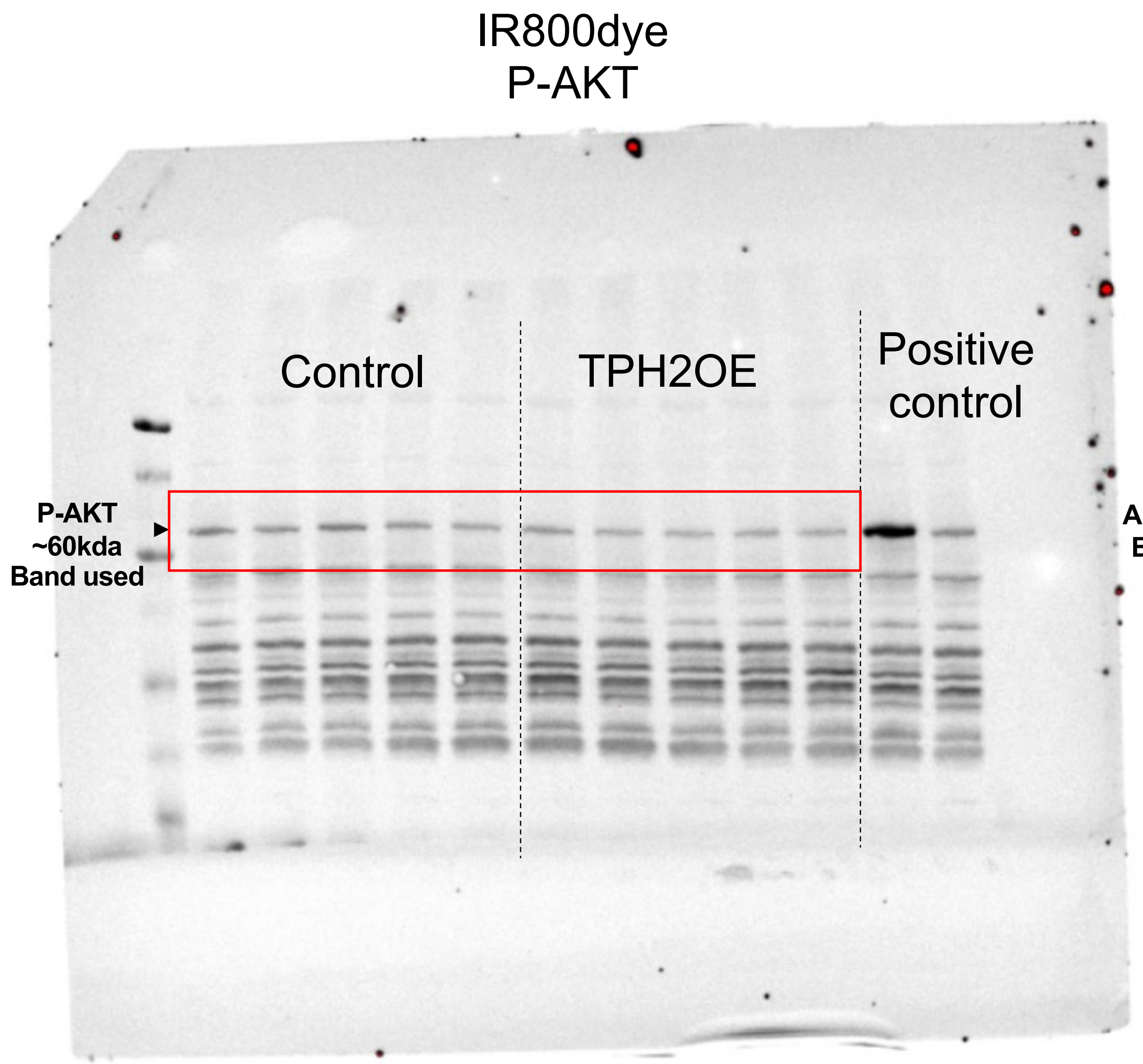

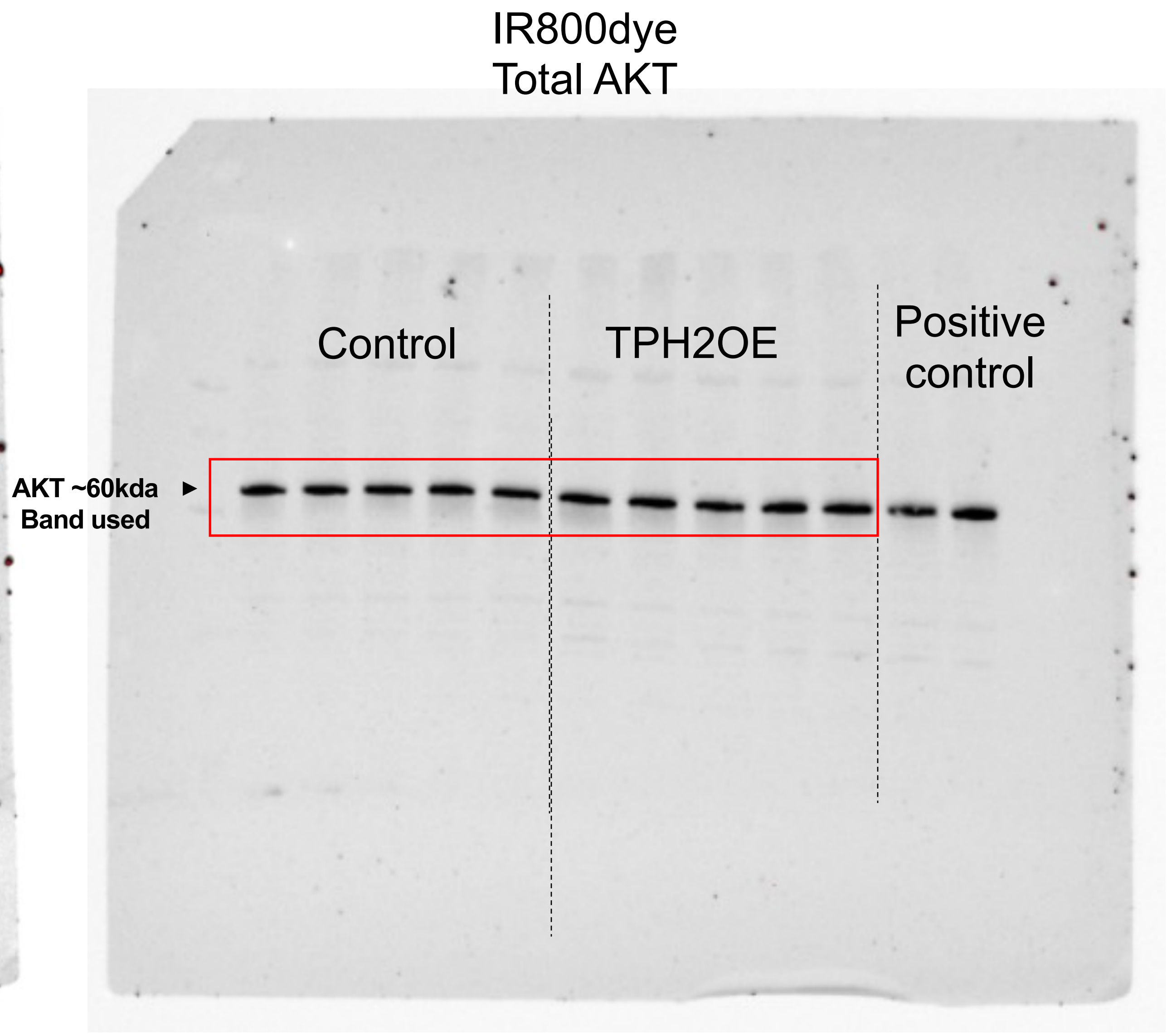

Full unedited blot for Figure 5R-eWAT

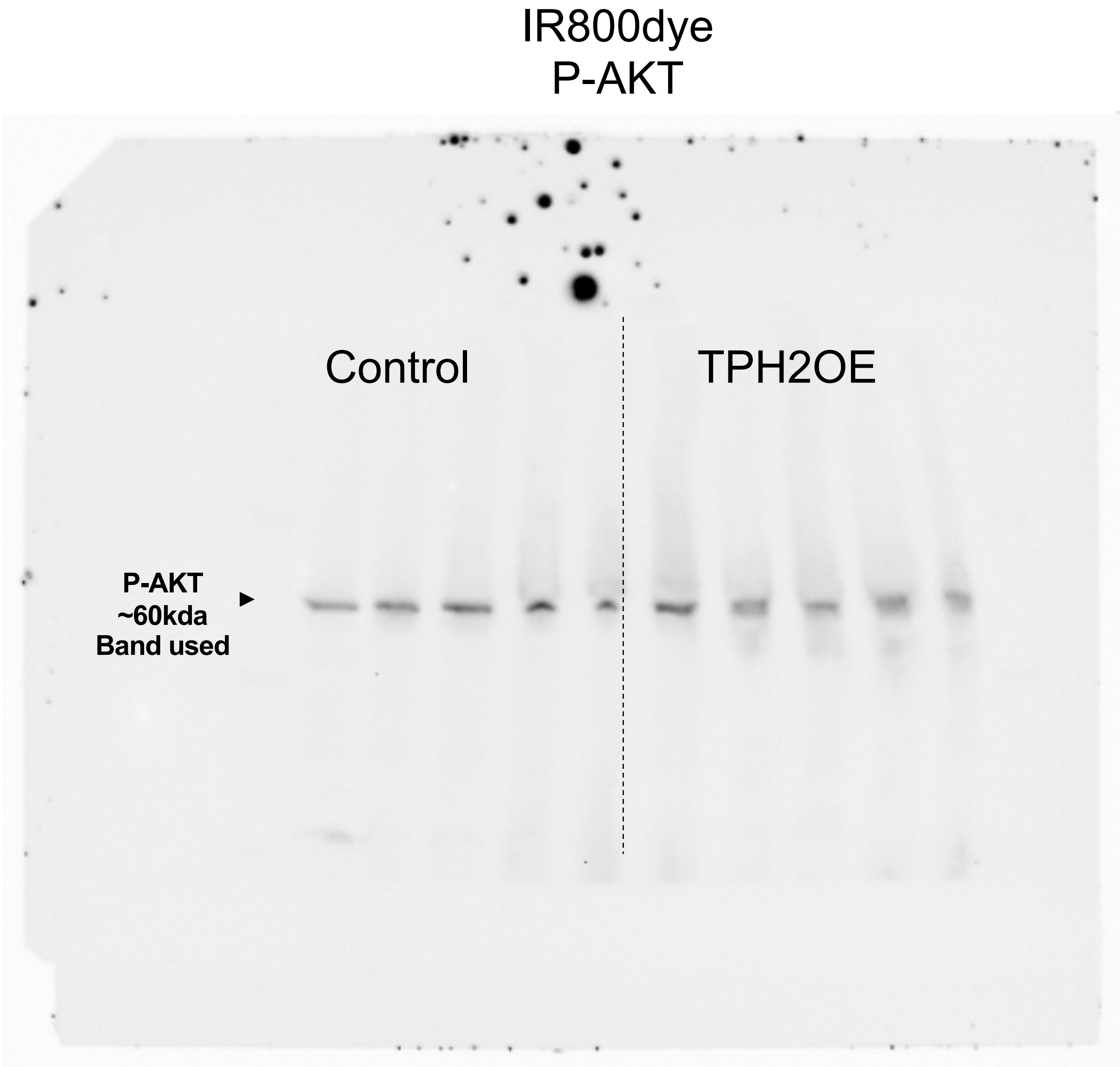

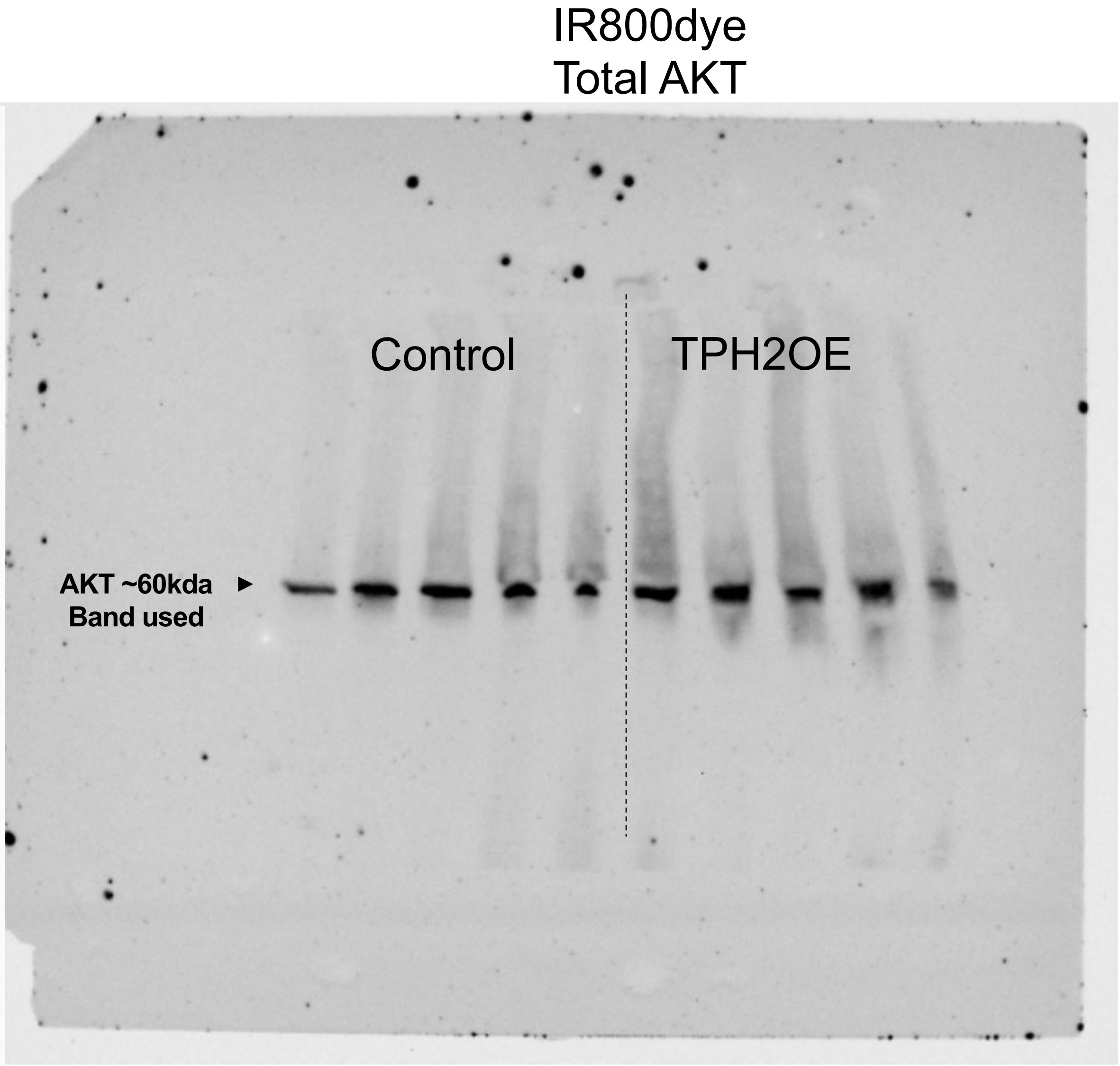

Full unedited blot for Figure 5R-Muscle

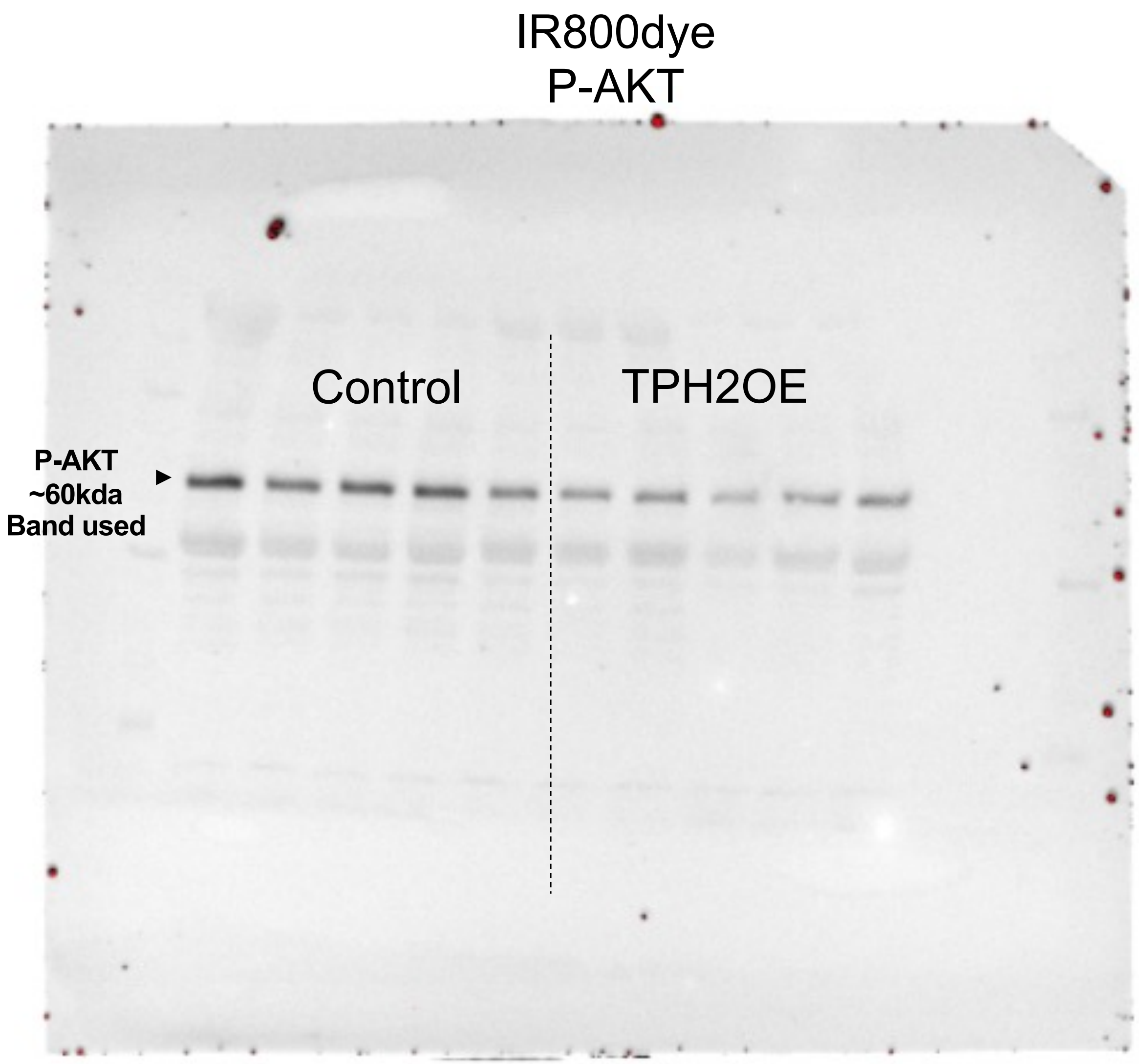

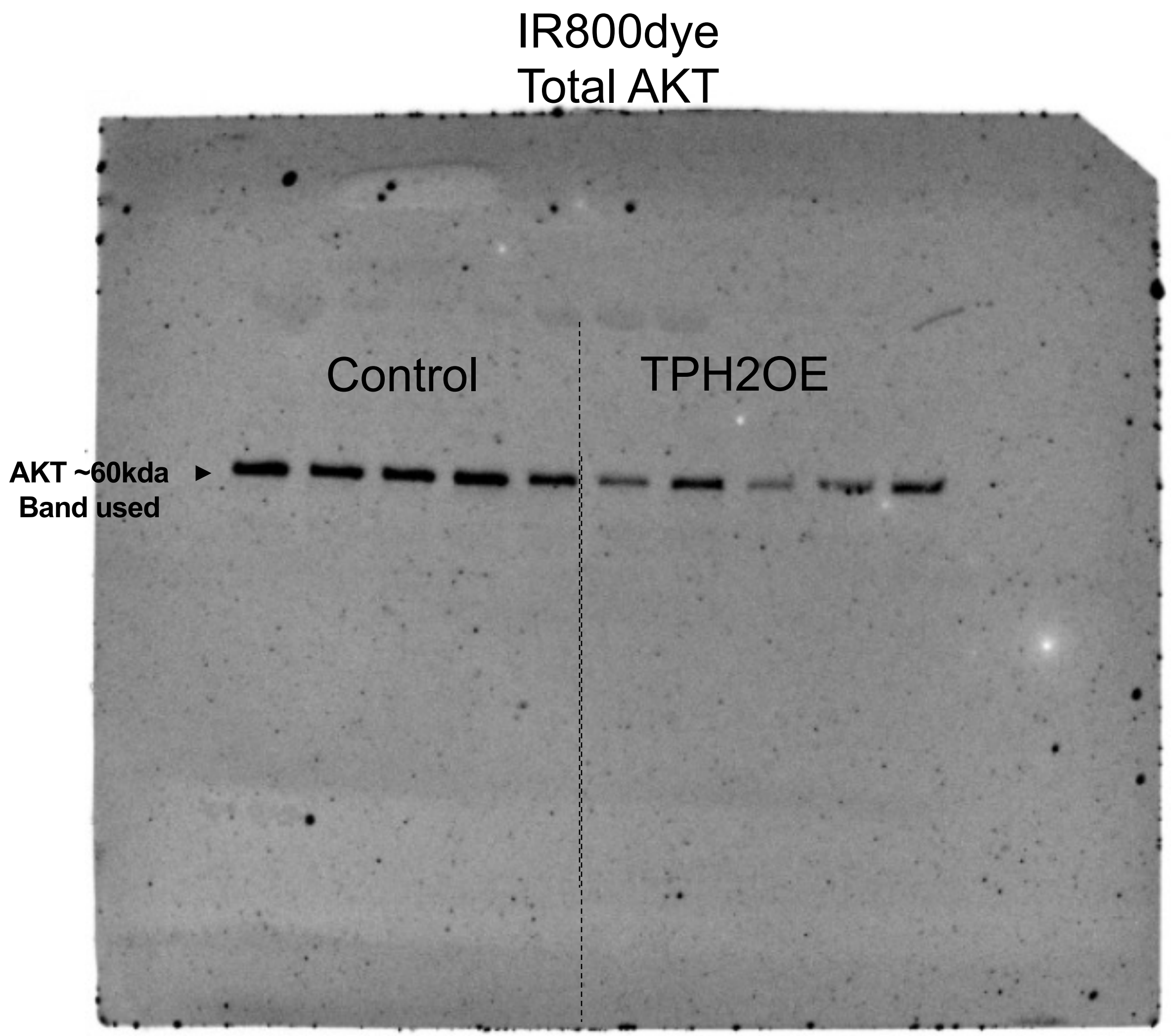

Full unedited blot for Figure 6I

IR800dye
TPH2

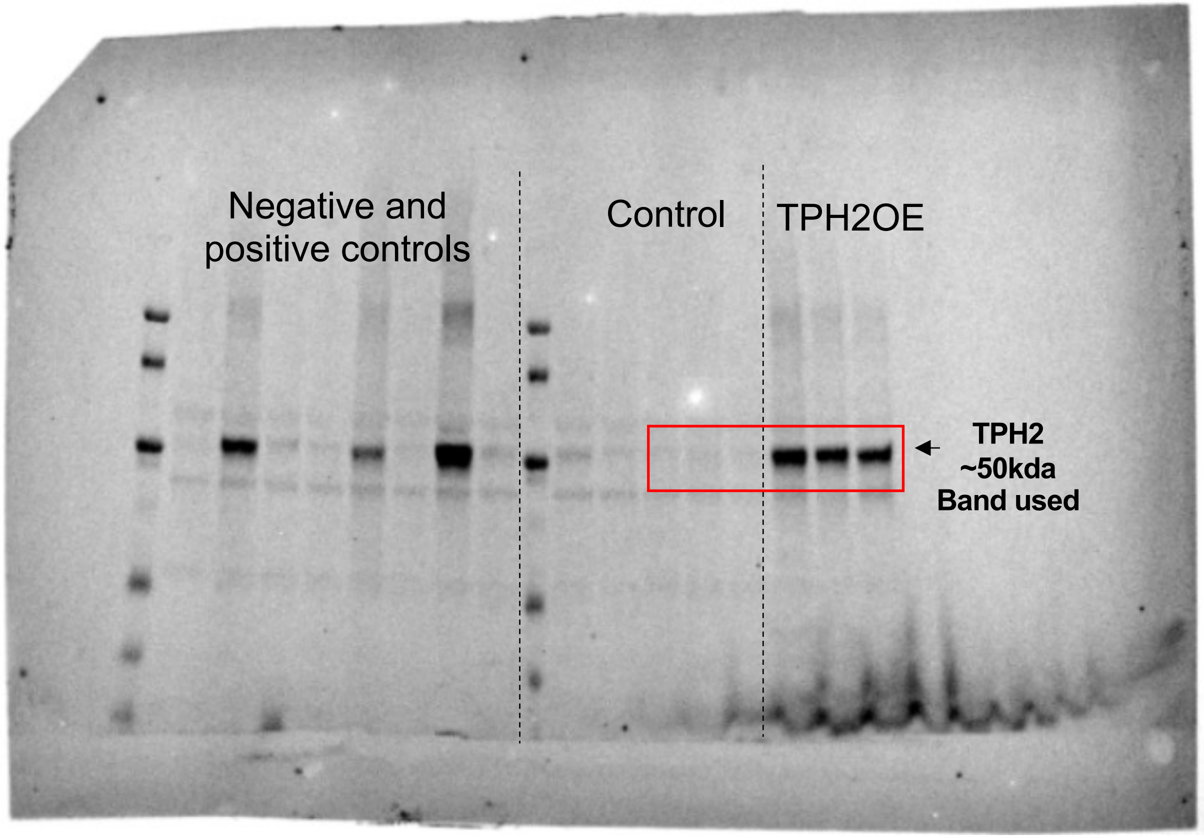

IR680dye
Beta actin

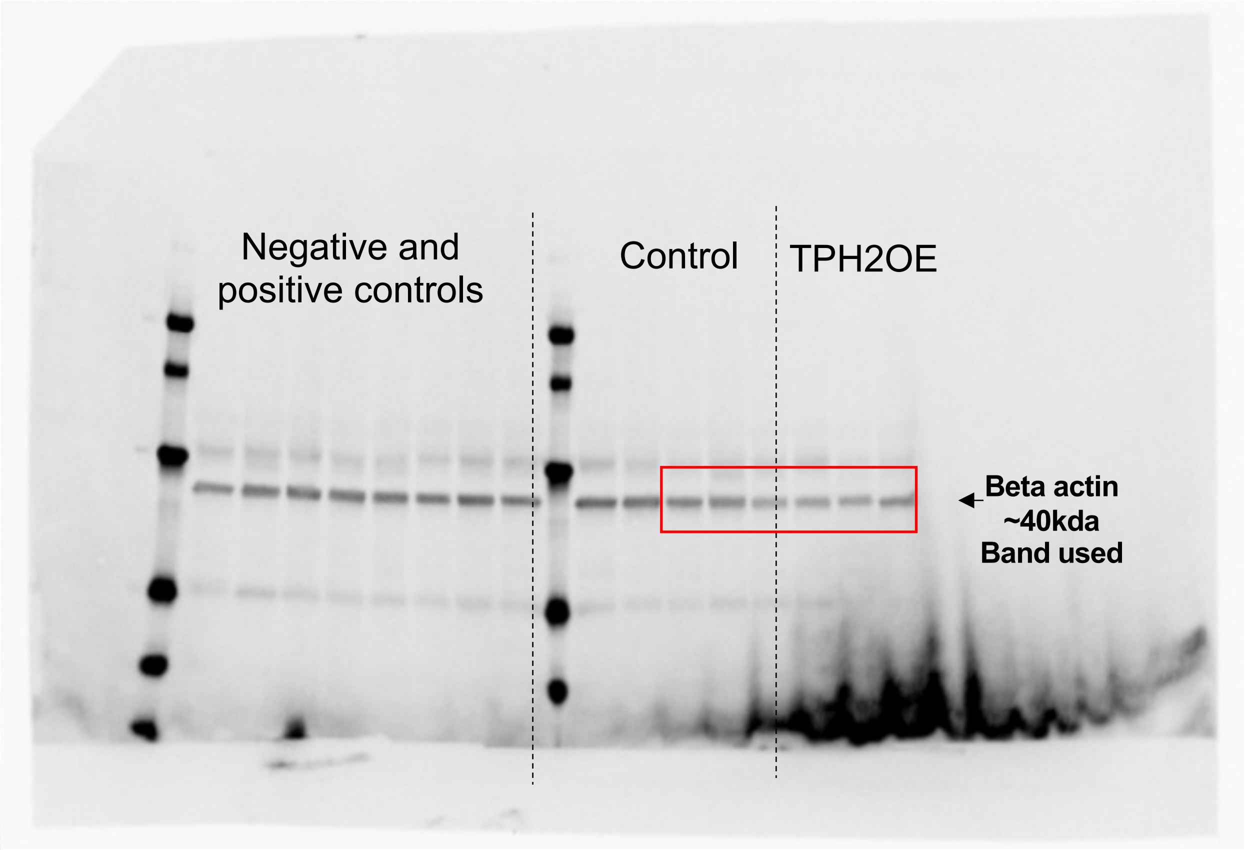

Full unedited blot for Figure 7G

IR800dye
UCP1

IR680dye
Beta actin

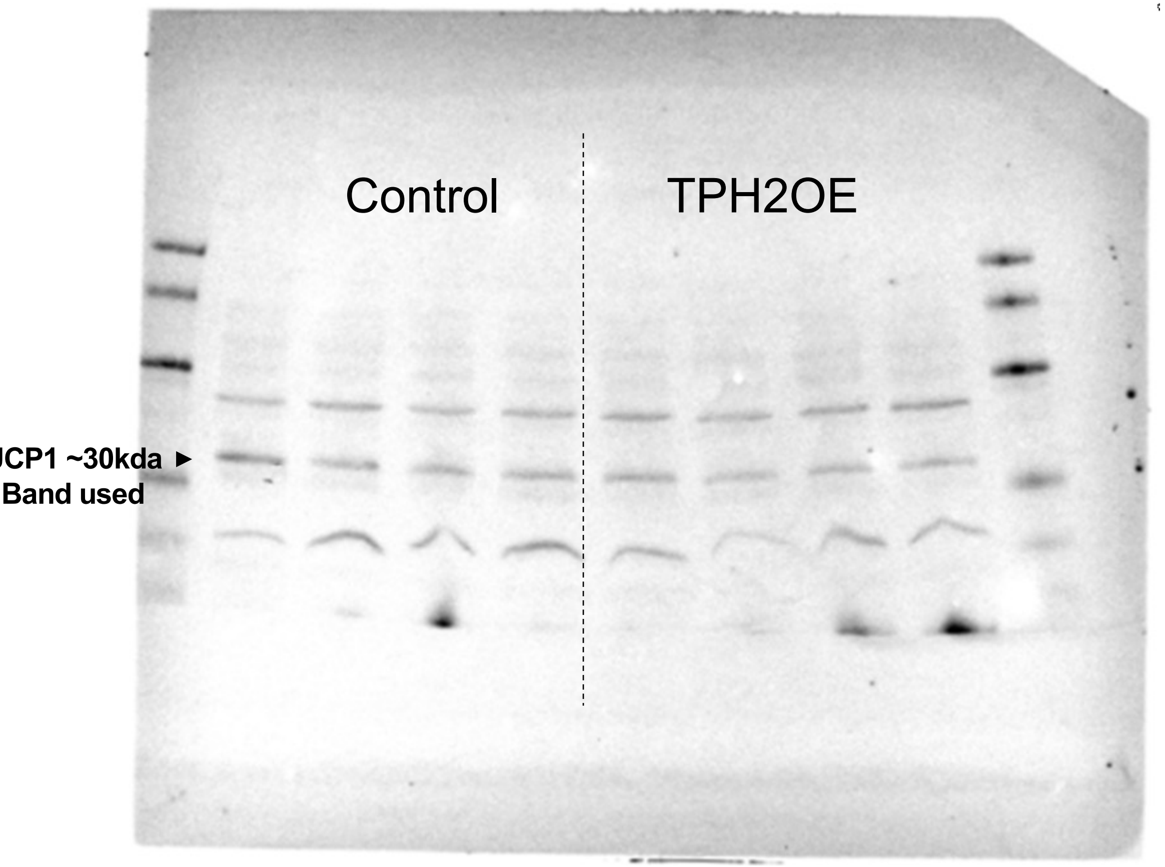

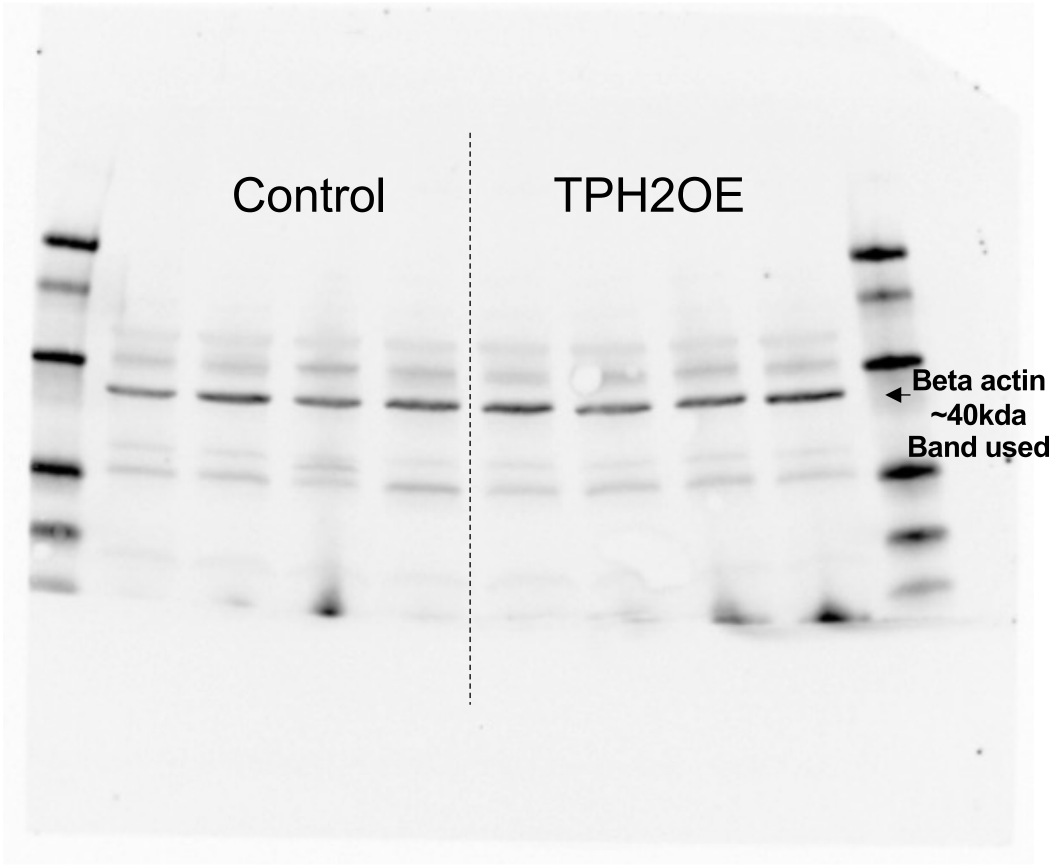

Full unedited blot for Figure 8G

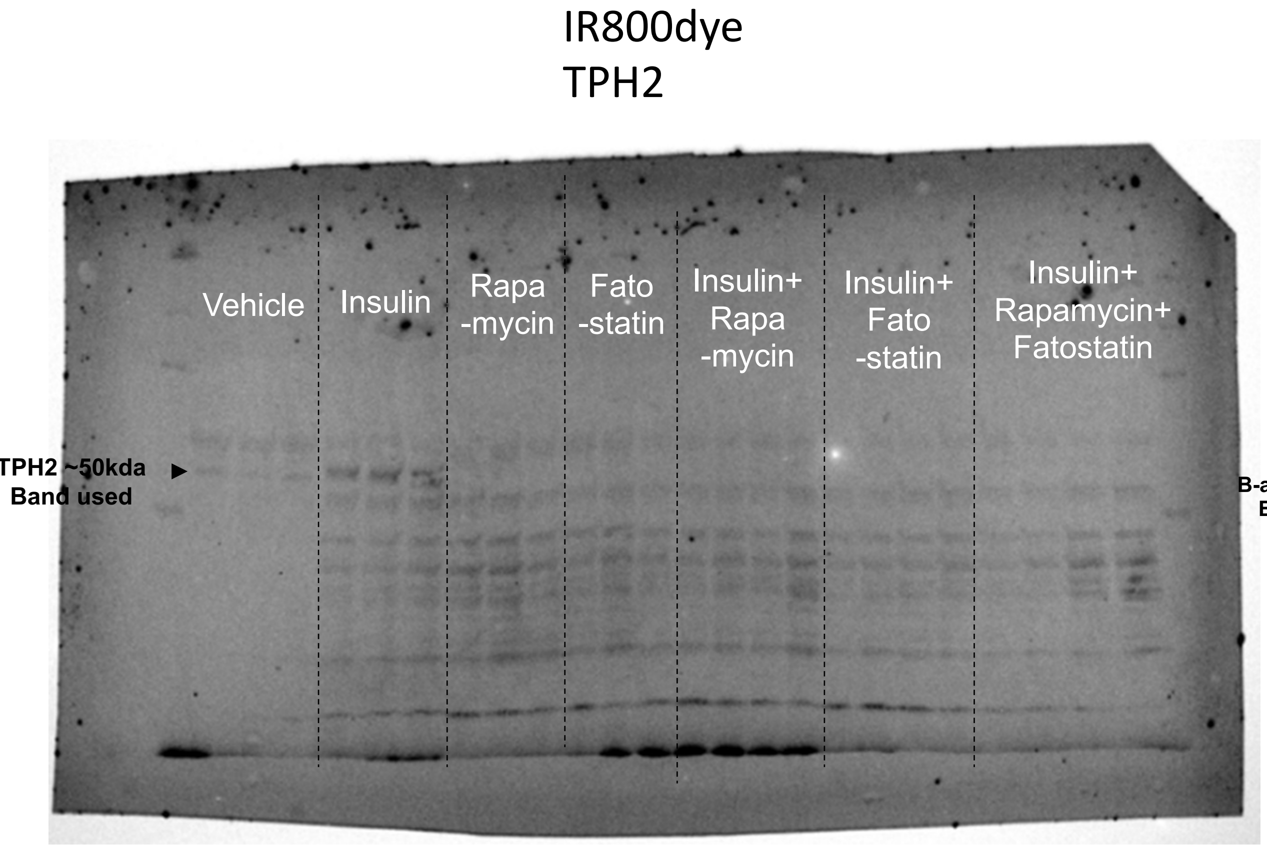

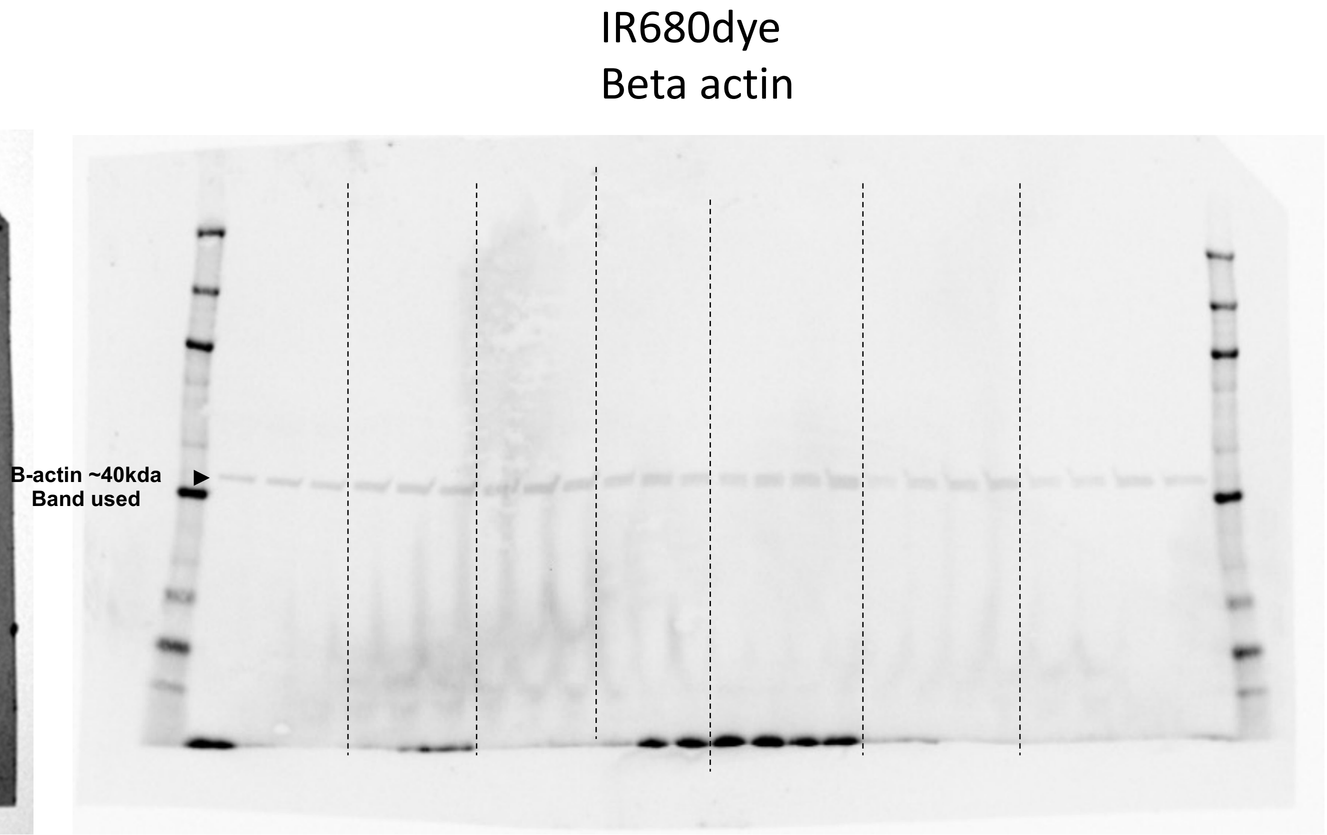

Supplement: Unedited blot and gel images [file jci-135-190765-s168.pdf]
